# *Kras* oncogene ablation prevents resistance in advanced lung adenocarcinomas

**DOI:** 10.1172/JCI164413

**Published:** 2023-04-03

**Authors:** Marina Salmón, Ruth Álvarez-Díaz, Coral Fustero-Torre, Oksana Brehey, Carmen G. Lechuga, Manuel Sanclemente, Fernando Fernández-García, Alejandra López-García, María Carmen Martín-Guijarro, Sandra Rodríguez-Perales, Emily Bousquet-Mur, Lucía Morales-Cacho, Francisca Mulero, Fátima Al-Shahrour, Lola Martínez, Orlando Domínguez, Eduardo Caleiras, Sagrario Ortega, Carmen Guerra, Monica Musteanu, Matthias Drosten, Mariano Barbacid

**Affiliations:** 1 Experimental Oncology Group, Molecular Oncology Program,; 2 Bioinformatics Unit,; 3 Molecular Cytogenetics and Genome Editing Unit, Human Cancer Genetics Program,; 4 Molecular Imaging Unit,; 5 Flow Cytometry Unit,; 6 Genomics Unit,; 7 Histopathology Unit, and; 8 Mouse Genome Editing Unit, Centro Nacional de Investigaciones Oncológicas (CNIO), Madrid, Spain.; 9 Centro de Investigación Biomédica en Red de Cáncer (CIBERONC), Instituto de Salud Carlos III, Madrid, Spain.; 10 Department of Biochemistry and Molecular Biology, Faculty of Pharmacy, Complutense University, Madrid, Spain.; 11 Molecular Mechanisms of Cancer Program, Centro de Investigación del Cáncer (CIC) and Instituto de Biología Molecular y Celular del Cáncer (IBMCC), Consejo Superior de Investigaciones Científicas–Universidad de Salamanca (CSIC-USAL), Salamanca, Spain.

**Keywords:** Oncology, Drug therapy, Lung cancer, Oncogenes

## Abstract

KRAS^G12C^ inhibitors have revolutionized the clinical management of patients with *KRAS*^G12C^-mutant lung adenocarcinoma. However, patient exposure to these inhibitors leads to the rapid onset of resistance. In this study, we have used genetically engineered mice to compare the therapeutic efficacy and the emergence of tumor resistance between genetic ablation of mutant *Kras* expression and pharmacological inhibition of oncogenic KRAS activity. Whereas *Kras* ablation induces massive tumor regression and prevents the appearance of resistant cells in vivo, treatment of *Kras*^G12C^/*Trp53*-driven lung adenocarcinomas with sotorasib, a selective KRAS^G12C^ inhibitor, caused a limited antitumor response similar to that observed in the clinic, including the rapid onset of resistance. Unlike in human tumors, we did not observe mutations in components of the RAS-signaling pathways. Instead, sotorasib-resistant tumors displayed amplification of the mutant *Kras* allele and activation of xenobiotic metabolism pathways, suggesting that reduction of the on-target activity of KRAS^G12C^ inhibitors is the main mechanism responsible for the onset of resistance. In sum, our results suggest that resistance to KRAS inhibitors could be prevented by achieving a more robust inhibition of KRAS signaling mimicking the results obtained upon *Kras* ablation.

## Graphical abstract

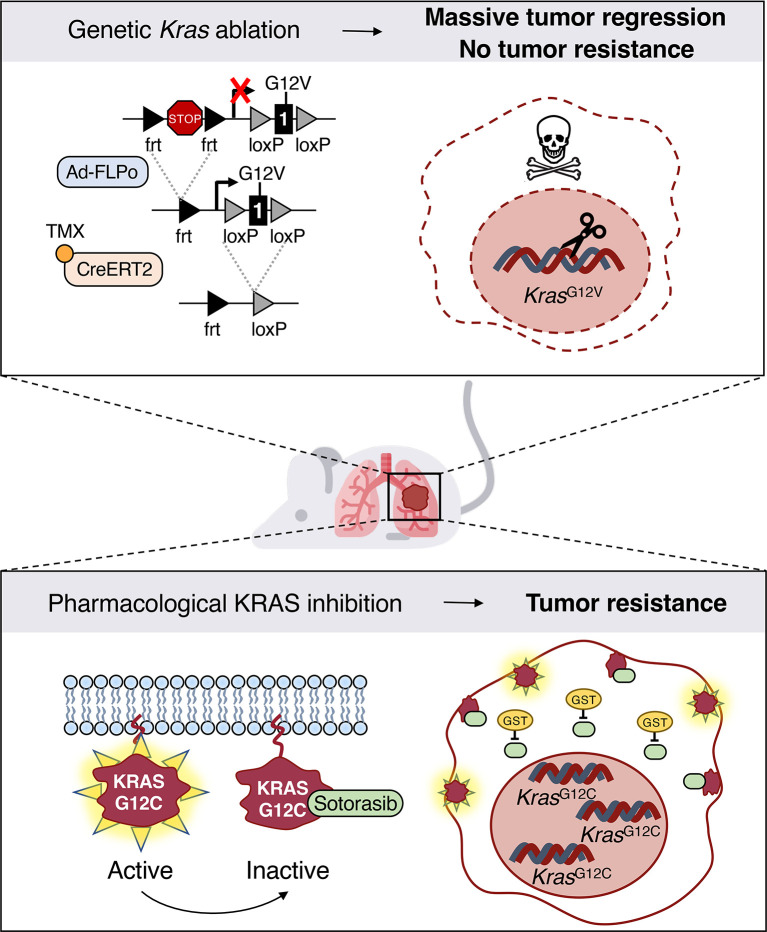

## Introduction

*KRAS* oncogenes have been identified in almost a quarter of all solid human tumors, including lung, colorectal, and pancreatic carcinomas, three tumor types with some of the lowest survival rates (1). Accumulating evidence indicates that their role in these diseases is not the same. Whereas *KRAS* oncogenes appear to be responsible for the initiation of lung and pancreatic adenocarcinomas, in colorectal tumors, they are involved in tumor progression, not initiation. Understanding the molecular bases for these differences as well as defining the precise role that *KRAS* oncogenes play not only in tumor progression, but in tumor maintenance, is a fundamental issue that needs to be properly addressed.

The differential roles that *KRAS* oncogenes play in human tumors have been considered as a mere academic issue with little or no impact in the clinic, since KRAS oncoproteins have been thought to be undruggable targets for over three decades. However, this concept is no longer accepted, since Shokat and his colleagues identified a previously unnoticed pocket located beneath the switch-II region of KRAS implicated in effector binding (2). This finding has stimulated the synthesis of compounds capable of occupying this space in a stable manner, provided that they could form a covalent bond with the mutant cysteine residue present at position 12 in the KRAS^G12C^ oncoprotein, the most frequent oncogenic *KRAS* variant in human lung adenocarcinomas (LUADs) (3). These compounds not only induce stabilization of the guanosine diphosphate–bound (GDP-bound) state of the oncoprotein, but also impede, at least partially, binding of the RAF effector proteins (2, 4, 5). Further improvement of the pharmacological properties of these compounds led to the design of potent inhibitors that could be approved by the FDA, such as sotorasib (AMG510), based on its efficacy in treating *KRAS*^G12C^ mutant lung tumors, as illustrated in the CodeBreaK100 phase I/II clinical trial (6). A second compound, adagrasib (MRTX849), was also granted accelerated approval very recently based on the results of the KRYSTAL phase I/II trial (7). Other KRAS^G12C^ inhibitors, such as JDQ443, characterized by a novel binding mode, have been described as well as new compounds that inhibit KRAS noncovalently and independently of its GDP/guanosine triphosphate (GTP) state (8, 9). Finally, a novel inhibitor, MRTX1133, selective for the *KRAS*^G12D^ mutation and capable of blocking the KRAS oncoprotein in its “on” state, has been described (10, 11).

Unfortunately, the clinical efficacy of KRAS^G12C^ inhibitors rapidly declines due to the development of resistance after just a few months of treatment (12–14). Molecular interrogation of resistant tumors has illustrated the presence of new mutations in the KRAS^G12C^ oncoprotein that prevented efficient inhibitor binding as well as mutations in the normal *KRAS* alleles that could not be targeted by sotorasib or adagrasib (13–15). Other tumors displayed mutations in upstream or downstream KRAS effectors. Interestingly, these novel mutations appear with low allelic frequency, raising the possibility that they may not be solely responsible for the observed tumor resistance (12–14). Whether those tumor cells carrying these new mutations will take over the entire tumor remains to be determined. Yet about half of the tumors examined did not display additional mutations, thus indicating the existence of additional mechanisms that might cause resistance to KRAS inhibition, at least in LUADs (16–19). Identification of these resistance mechanisms is an urgent prerequisite to developing improved therapeutic strategies that may delay or even prevent the emergence of resistance.

In the present study, we have used genetic approaches for illustrating the essential role of *Kras* oncogene expression in tumor maintenance as well as in the appearance of resistant cells, both in vitro and in vivo. In addition, we have generated pharmacologically resistant tumors using an experimental model of *Kras*^G12C^/*Trp53*–driven LUAD in an effort to shed light on the potential mechanisms responsible for the appearance of resistance in a clinical scenario.

## Results

### Kras oncogene ablation induces massive regression of advanced Kras^G12V^/Trp53-driven LUADs.

We first investigated whether continuous expression of the *Kras* oncogene was essential for tumor progression and maintenance as well as for the appearance of resistant tumor cells. To this end, we generated a strain of mice that carries a floxed *Kras*^FSFG12V^ allele, *Kras*^FSFG12Vlox^, in which we could eliminate expression of the KRAS oncoprotein by Cre-mediated recombination (Supplemental Figure 1; supplemental material available online with this article; https://doi.org/10.1172/JCI164413DS1; see also supplemental material for full, uncut gels). Infection of *Kras*^+/FSFG12Vlox^*;Trp53*^F/F^ mice (designated K^G12Vlox^P) with Adeno-FLPo led to the development of lung tumors indistinguishable from those previously observed in the *Kras*^+/FSFG12V^*;Trp53*^F/F^ strain. Ablation of the resulting *Kras*^G12Vlox^ allele was subsequently achieved by expressing 2 independent *CreERT2* alleles, such as the *hUBC-CreERT2* transgene and the endogenous *Rosa26-CreERT2* allele, followed by tamoxifen (TMX) exposure. Expression of both *CreERT2* alleles was essential for effectively reducing the number of recurrent tumors due to the expression of unrecombined *Kras*^G12Vlox^ alleles.

*Kras*^+/FSFG12Vlox^*;Trp53*^F/F^*;Rosa26-CreERT2*^KI/KI^*;Tg.hUBC-CreERT2*^+/T^ mice (designated as K^G12Vlox^PC2) were exposed to Adeno-FLPo particles to induce lung tumors. Mice bearing, on average, 2 tumors per animal that could be effectively monitored by CT scans were exposed to a TMX diet to activate the resident CreERT2 recombinases and enrolled in a preclinical trial to determine the fate of their lung tumors. A limited number of mice carrying progressive tumors due to incomplete recombination of the *Kras*^G12Vlox^ allele were not included in the study.

**Figure 1:**
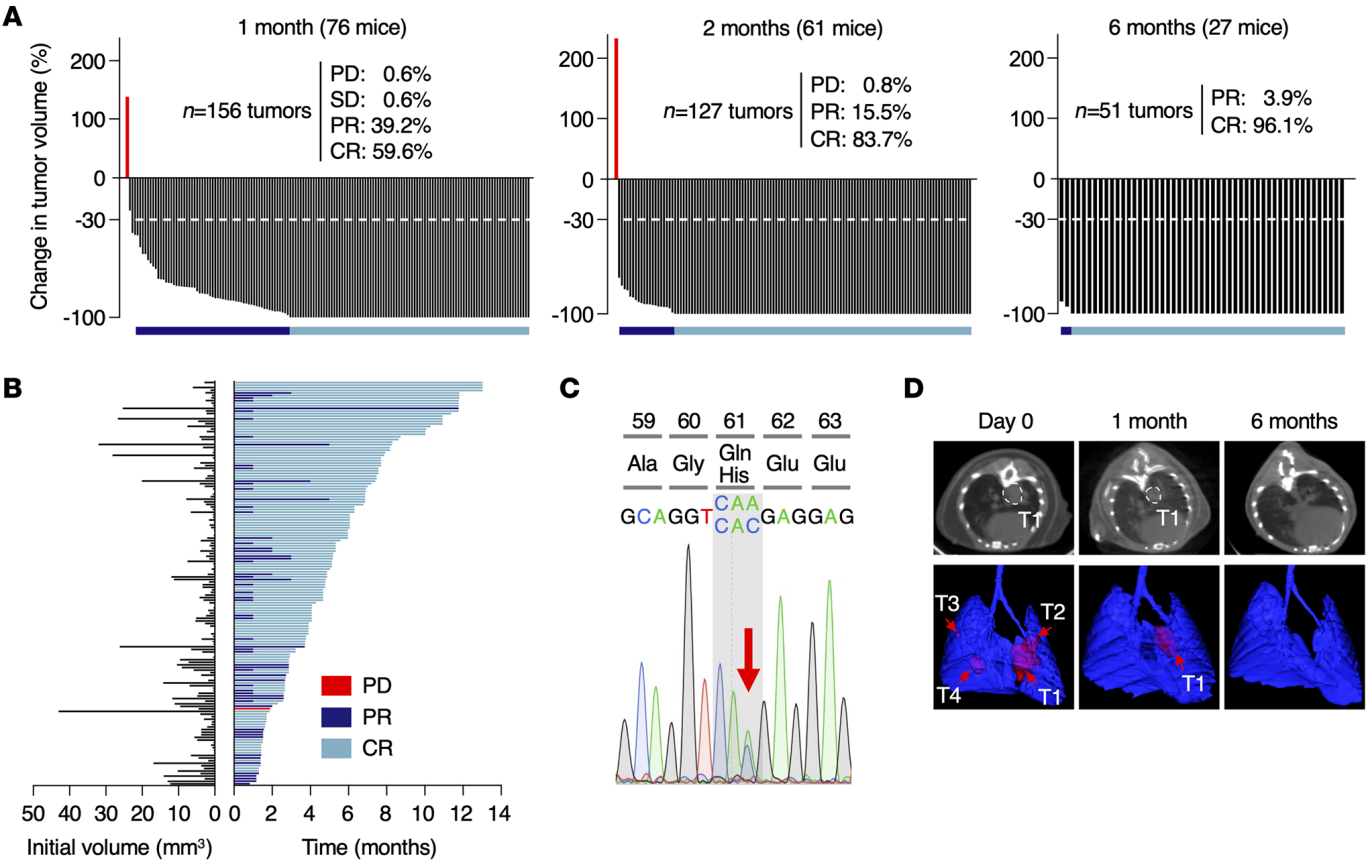
Genetic ablation of *Kras*^G12V^ in K^G12Vlox^PC2 lung tumors induces massive tumor regression. (**A**) Waterfall plots representing the changes in tumor volume of tumors present in K^G12Vlox^PC2 mice exposed to a TMX diet for 1 (*n* = 76 mice/156 tumors), 2 (*n* = 61 mice/127 tumors), and 6 months (*n* = 27 mice/51 tumors), as determined by CT scans. Percentages of tumors showing progressive (PD) or stable disease (SD), PR, or CR are depicted in the figure. A growing tumor lacking the resident *Kras*^G12V^ oncogene is depicted in red. The dotted lines mark 30% regression levels. Horizontal bars indicate tumors undergoing PR (dark blue) and CR (light blue). (**B**) Initial tumor size (left) and duration of response (right) from individual tumors represented in **A** until they reach a humane end point. Colors are those described in **A**. (**C**) Sequencing chromatogram depicting the Q61H mutation in the WT *Kras* allele present in the single tumor that displayed PD after *Kras*^G12V^ ablation. The arrow indicates the WT (CAA) and mutated (CAC) codons. (**D**) Representative images illustrating CT scans (top) and 3D rendering (bottom) of lungs depicting tumor response after 1 and 6 months of TMX exposure. Tumors are outlined (top) or indicated by arrows (bottom). T1, tumor 1.

As illustrated in Figure 1A, exposure of 76 K^G12Vlox^PC2 mice harboring 156 lung tumors ranging in size from 0.13 to 43 mm^3^ to the TMX diet for just 1 month led to the complete regression (CR), based on negative CT scans, of almost two-thirds of the tumors (93 out of 156, 59.6%) (Figure 1A). Equally important was the significant reduction (>30% in tumor volume) observed in 61 out of the 156 tumors (39.2%), considered as partial regression (PR). Moreover, the relative levels of tumor regression were independent of the original size of the tumor, and even large tumors (>40 mm^3^) displayed CRs (Figure 1B).

Tumor regression was even more evident when mice were examined after 2 months of TMX exposure. As illustrated in Figure 1A, the percentage of CRs increased to 83.7% (106 out of 127 tumors), while those displaying PRs greater than 30% accounted for the rest of the tumors (20 out of 127, 15.5%), except for a single tumor that continued expanding in size during this 2-month period, until the corresponding mouse had to be sacrificed (Figure 1A). Molecular analysis of this tumor revealed that the *Kras*^G12Vlox^ allele was completely excised, indicating that it was a bona fide *Kras*-resistant tumor. Whole-exome sequencing (WES) analysis revealed the presence of a single A-to-C transversion that led to a Q61H miscoding mutation located in the normal *Kras* allele (Figure 1C). Q61H mutations are known to activate the transforming properties of *Kras* and have been observed in human tumors resistant to adagrasib (13).

Tumor monitoring was extended for 6 months of TMX exposure (Figure 1, A and D). At this time point, only 2 mice carried CT^+^ tumors. Mice surviving the 6-month time point were allowed to thrive until the time when they had to be sacrificed at a humane end point. Few mice survived beyond 10 months of TMX exposure. Interestingly, one of these mice carried a tumor that did not completely regress after up to 1 year of treatment. The molecular bases for the long latency of a small fraction of tumors (<2.5%) before they completely regressed remains to be determined. Although we did not observe overt toxicity after 12 months on a continuous TMX diet (Supplemental Figure 2, A and B), a significant proportion of K^G12Vlox^PC2 mice died prematurely due to causes unrelated to lung tumors, such as skin ulcers (Supplemental Figure 2B). Pathological examination of the lungs of these mice also failed to reveal the presence of lung tumors. Thus, ablation of *Kras* oncogene expression in advanced lung tumors not only induced massive tumor regression, but also prevented the appearance of resistant tumors, except for the spontaneous *Kras*^Q61H^ allele described above.

### Kras oncogene ablation induces apoptosis and remodeling of the tumor microenvironment in vivo.

To define the mechanism underlying tumor regression upon *Kras*^G12Vlox^ ablation, tumor-bearing K^G12Vlox^PC2 mice were exposed to a TMX-containing diet for 1 or 2 weeks and samples were collected for further analysis. Histological examination revealed a significant decline in Ki67^+^ as well as pERK^+^ tumor cells, indicative of reduced proliferation (Supplemental Figure 3, A and B). More importantly, *Kras*^G12Vlox^ ablation resulted in a strong increase in the number of apoptotic cells after 1 week on the TMX diet, along with a progressing reduction in tumor grades (20) (Supplemental Figure 3, A–C). In addition, we detected growing numbers of CD8^+^ T cell infiltrates after 1 and 2 weeks on the TMX diet and a significant increase in NK cells (Supplemental Figure 3D). No change in CD4^+^ T cells or F4/80^+^ macrophages could be detected (Supplemental Figure 3D).

To functionally validate the contribution of T cells to tumor regression upon *Kras*^G12V^ ablation, we subcutaneously injected 2 independent K^G12Vlox^PC2 tumor cell lines (see below) into athymic NU-Foxn1^nu^ mice unable to produce T cells and exposed established tumors to the TMX diet. As depicted in Supplemental Figure 3E, *Kras*^G12Vlox^ ablation in the absence of T cells still prevented tumor progression, but did not result in rapid tumor regression, thus indicating that T cells contributed to inducing tumor regression. Together, these data indicate that apoptosis is the most immediate response to *Kras*^G12V^ ablation in tumors from K^G12Vlox^PC2 mice, followed by subsequent remodeling events in the tumor microenvironment that partially contribute to tumor regression.

### Urethane-induced tumors regress upon Kras oncogene ablation.

**Figure 2:**
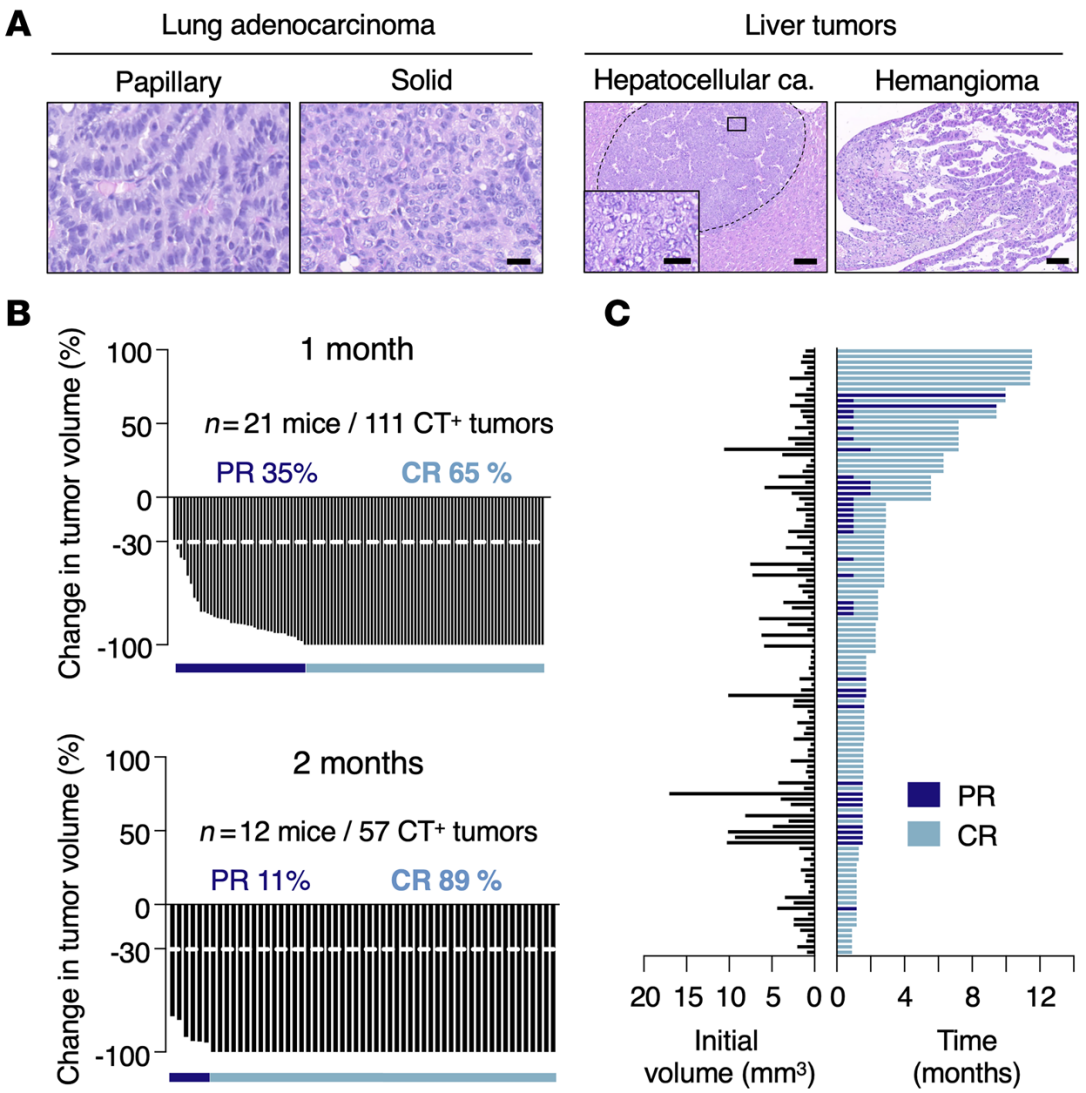
Effect of *Kras* ablation in urethane-induced lung tumors. (**A**) Representative images of H&E-stained, paraffin-embedded sections of LUADs with papillary or solid structure (left) and of liver tumors including a hepatocellular carcinoma and a hemangioma (right) present in K^lox^PC2 mice exposed to urethane. Scale bars: 100 μm (low magnification); 20 μm (high magnification). (**B**) Waterfall plots representing changes in tumor volume in K^lox^PC2 mice exposed to a TMX diet for 1 month (*n* = 21 mice/111 tumors) (top) or 2 months (*n* = 12 mice/57 tumors) (bottom). Percentages of tumors undergoing PR (dark blue bar) or CR (light blue bar) are depicted in the figure. (**C**) Initial tumor size (left) and duration of response (right) from individual tumors represented in **B**. Colors indicate whether individual tumors underwent PR (dark blue) or CR (light blue) up to the time of the humane end point.

We also interrogated the therapeutic effect of *Kras* oncogene ablation in tumors induced by urethane, a chemical carcinogen known to induce lung tumors with *Kras* mutations, preferentially at codon 61 (21, 22). To this end, we used a *Kras* conditional (floxed) strain previously developed in the laboratory to which we added the *CreERT2* loci described above. The resulting mice, *Kras*^lox/lox^;*Rosa26-CreERT2*^KI/KI^;*Tg.hUBC-CreERT2*^+/T^ (referred to as K^lox^C2), were exposed to a single dose of urethane (1 g/kg) at 4 weeks of age. Urethane-induced tumors appeared with longer latencies (47 weeks after urethane injection) than those induced in the genetically engineered K^G12Vlox^PC2 mouse tumor model (30 weeks after Adeno-FLPo exposure). *Kras* codon 61 mutations were detected in all cases analyzed (*n* = 11) (21). Of these tumors, 8 exhibited CAA>CGA transitions (Q61R), and 3 displayed CAA>CTA transversions (Q61L). Histopathological examination of these tumors revealed formation of adenocarcinomas with papillary-solid architecture, varying in the degree of malignancy (Figure 2A).

Once tumors were detected by CT analysis, K^lox^C2 mice (*n* = 21, 111 CT^+^ tumors) were subjected to continuous TMX diets to mediate recombination of the *Kras*^lox^ alleles. As illustrated in Figure 2, B and C, *Kras* ablation dramatically decreased tumor burden after 1 month of TMX exposure, reaching 65% CRs and 35% PRs, a result similar to that observed in the K^G12Vlox^PC2 tumor model. Likewise, tumor regression was independent of the initial size of the tumor (0.3 to 17 mm^3^) (Figure 2C). Extended exposure to the TMX diet for an additional month resulted in an increased percentage of tumors undergoing CRs (89%). No progressive or stable disease was identified in this trial. Moreover, none of the 12 surviving K^lox^C2 mice allowed to thrive displayed signs of tumor relapse, including those (*n* = 7) that survived more than 4 months (Figure 2C). Indeed, histological examination of urethane-treated K^lox^C2 mice sacrificed upon a humane end point failed to reveal detectable lung tumors. These results illustrate that the massive rate of tumor regressions, as well as the absence of resistant tumors observed upon *Kras* oncogene ablation, were not limited to the genetically engineered K^G12Vlox^PC2 tumor model. Finally, about a third of the treated K^lox^C2 mice developed pathologies related to urethane administration, such as eye ulcers, polyps in the colon, hemangiomas, hepatic peliosis, and, to a lesser extent, hepatocellular carcinomas (Figure 2A). These toxic effects along with the relative advanced age of the mice and the long-term exposure to TMX are likely to contribute to the premature death of the treated mice.

### Cell-autonomous resistance to Kras oncogene expression is mediated by NF-κB and STAT3 signaling.

**Figure 3:**
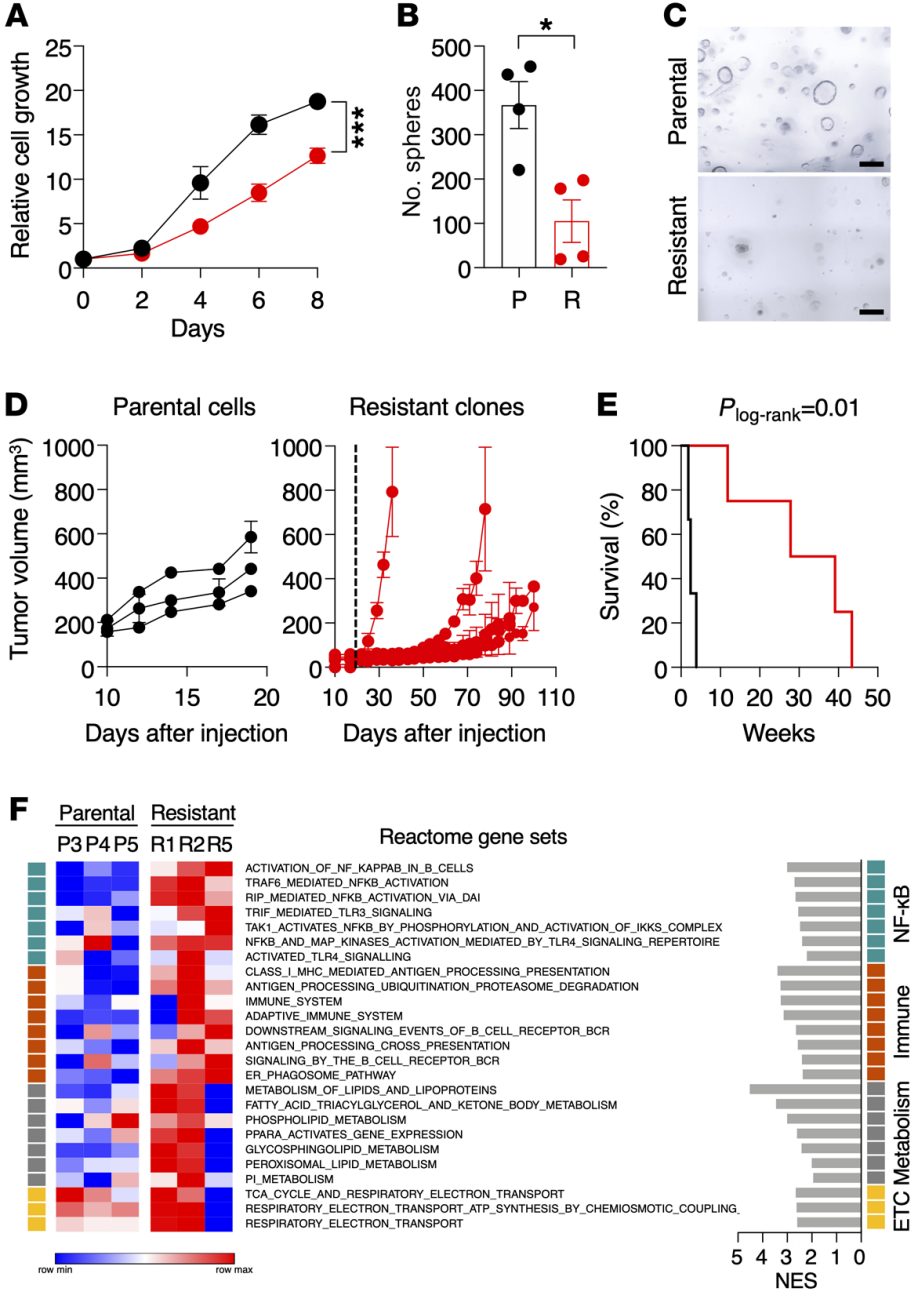
Characterization of tumor cells resistant to genetic *Kras*^G12V^ ablation. (**A**) Relative growth of *Kras*^+/G12Vlox^;*Trp53^–/–^* tumor cells (black circles, *n* = 5) derived from K^G12Vlox^P mice and resistant *Kras^+/–^*;*Trp53^–/–^* clones, obtained upon ablation of the *Kras*^G12V^ oncogene (red circles, *n* = 9) for the indicated times. Data are represented as mean ± SEM. *P* values were calculated using unpaired Student’s *t* test by comparing areas under the curve. ***P < 0.001. (**B**) Quantification of 3D spheres of parental *Kras*^+/G12Vlox^;*Trp53^–/–^* cells (P, black circles, *n* = 4) and resistant *Kras^+/–^*;*Trp53^–/–^* clones (R, red circles, *n* = 4) in Matrigel for 7 days. Data are represented as mean ± SEM. *P* values were calculated using unpaired Student’s *t* test. *P < 0.05. (**C**) Representative images of 3D spheres of parental *Kras*^+/G12Vlox^;*Trp53^–/–^* cells and resistant *Kras^+/–^*;*Trp53^–/–^*clones grown in Matrigel for 7 days. Scale bars: 200 μm. (**D**) Tumor growth of parental cell lines and resistant clones after subcutaneous implantation in immunodeficient mice. Each lane represents an independent *Kras*^+/G12Vlox^;*Trp53^–/–^* cell line (black circles, *n* = 3) (left) or resistant *Kras^+/–^*;*Trp53^–/–^* clone (red circles, *n* = 6) (right). Dotted line marks the maximum time we allowed parental cells to grow (20 days) for comparison purposes. Data are represented as mean ± SEM. (**E**) Survival of immunodeficient mice after transpleural orthotopic injection of parental *Kras*^+/G12Vlox^;*Trp53^–/–^* cell lines (black, *n* = 3) and resistant *Kras^+/–^*;*Trp53^–/–^* clones (red, *n* = 4). *P_log-rank_* = 0.01. (**F**) Heatmap representing color-coded enrichment scores from single-sample GSEA analysis of RNA-Seq data using Reactome gene sets comparing 3 parental *Kras*^+/G12Vlox^;*Trp53^–/–^* cell lines and 3 resistant *Kras^+/–^*;*Trp53^–/–^* clones. Gene sets were ranked based on related functions indicated on the right. The normalized enrichment score (NES) is also shown. Only gene sets significantly enriched at FDR *q* values < 0.25 were considered.

Next, we interrogated the existence of cell-autonomous pathways that could play a role in the development of resistance to KRAS inhibition (23). To this end, we explanted tumors derived from K^G12Vlox^P mice to generate cell lines. Tumor cells were infected with Adeno-Cre particles to excise the floxed sequences. Ablation of *Kras*^G12V^ oncogene expression induced abundant apoptosis (Supplemental Figure 4), yet a limited number of cells survived and were able to proliferate under standard culture conditions. These cells displayed the expected *Kras^+/–^*;*Trp53^–/–^* genotype (Supplemental Figure 5A), proliferated less than the corresponding parental *Kras*^+/G12Vlox^;*Trp53^–/–^* cells, and formed significantly fewer 3D spheres (Figure 3, A–C). Yet they were able to form tumors in immunocompromised mice when injected subcutaneously (Figure 3D). They also induced tumors when injected orthotopically into the lungs, albeit with a much longer latency than the parental controls (Figure 3E). Thus, ablation of the resident *Kras* oncogene in these cells did not eliminate their tumorigenic properties.

Western blot and mass spectrometric analysis demonstrated no changes in the expression levels of the normal KRAS protein in 7 out of 8 resistant *Kras^+/–^*;*Trp53^–/–^* clones (Supplemental Figure 5, B–D). Likewise, the levels of expression of the other RAS paralogs, NRAS and HRAS, also remained unaltered (Supplemental Figure 5, B–D). One *Kras^+/–^*;*Trp53^–/–^* resistant clone displayed elevated levels of KRAS as well as total GTP-bound RAS (Supplemental Figure 5B), suggesting that amplification of the WT KRAS protein may represent a strategy for restoring KRAS activity upon *Kras* oncogene elimination.

**Figure 4:**
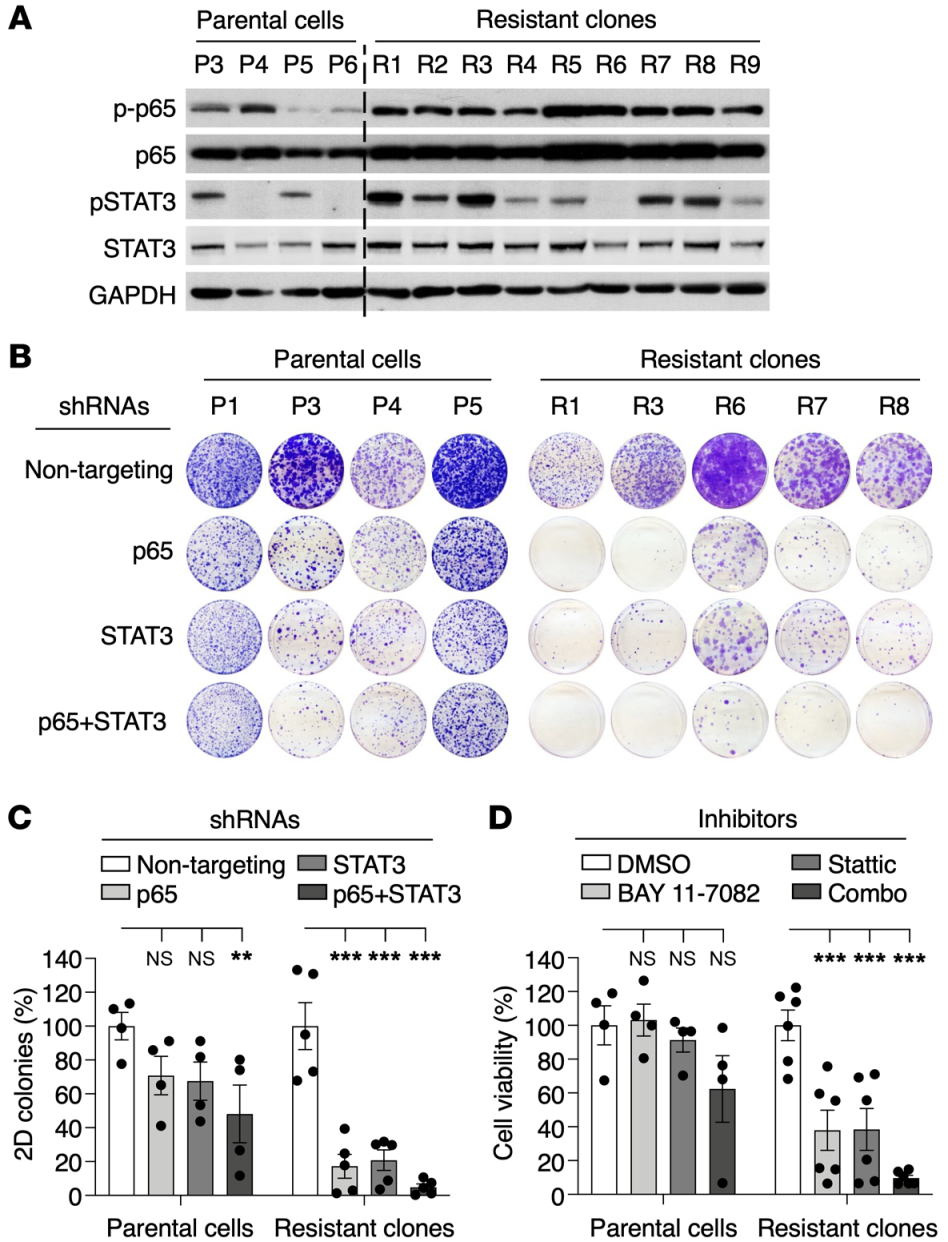
NF-κB and STAT3 signaling mediate survival of resistant *Kras^+/–^;Trp53^–/–^* clones. (**A**) Western blot analysis of phospho-p65 (p-p65), p65, p-STAT3, and STAT3 expression in parental *Kras*^+/G12Vlox^;*Trp53^–/–^* cell lines (P3 to P6) and resistant *Kras^+/–^*;*Trp53^–/–^* clones (R1 to R9). GAPDH served as loading control. (**B**) Colony formation assays on 10 cm cell culture dishes of parental *Kras*^+/G12Vlox^;*Trp53^–/–^* cell lines and resistant *Kras^+/–^*;*Trp53^–/–^* clones expressing either nontargeting shRNA or shRNAs against p65 and/or STAT3. (**C**) Quantification of the number of colonies present in the experiment described in **B**. Data are represented as mean ± SEM. *P* values were calculated using 2-way ANOVA. **P < 0.01; ***P < 0.001. (**D**) Viability of parental *Kras*^+/G12Vlox^;*Trp53^–/–^* cell lines (*n* = 4) and resistant *Kras^+/–^*;*Trp53^–/–^* clones (*n* = 6) treated with BAY 11-7082 (10 μM) and Stattic (5 μM) either individually or in combination (Combo). Data are represented as mean ± SEM. *P* values were calculated using 2-way ANOVA. ***P < 0.001.

To identify the pathways responsible for the tumorigenic properties of those cells lacking *Kras*^G12V^ expression, we submitted resistant *Kras^+/–^*;*Trp53^–/–^* clones along with parental *Kras*^+/G12Vlox^;*Trp53^–/–^* cell lines to RNASeq analysis (Supplemental Table 1). As shown in Figure 3F, resistant cells displayed substantial upregulation of gene sets related to NF-κB signaling or activation of immune pathways, and, to a lesser extent, several gene sets linked to metabolism or electron transport in mitochondrial respiration. Western blot analysis of resistant clones confirmed overt activation of NF-κB signaling, as demonstrated by phosphorylation and increased nuclear localization of p65 (RELA) (Figure 4A and Supplemental Figure 6). We also detected increased phosphorylation and nuclear localization of STAT3 in most, albeit not all, clones, thus adding further support to the results obtained by RNA-Seq (Figure 4A and Supplemental Figure 6).

**Figure 5:**
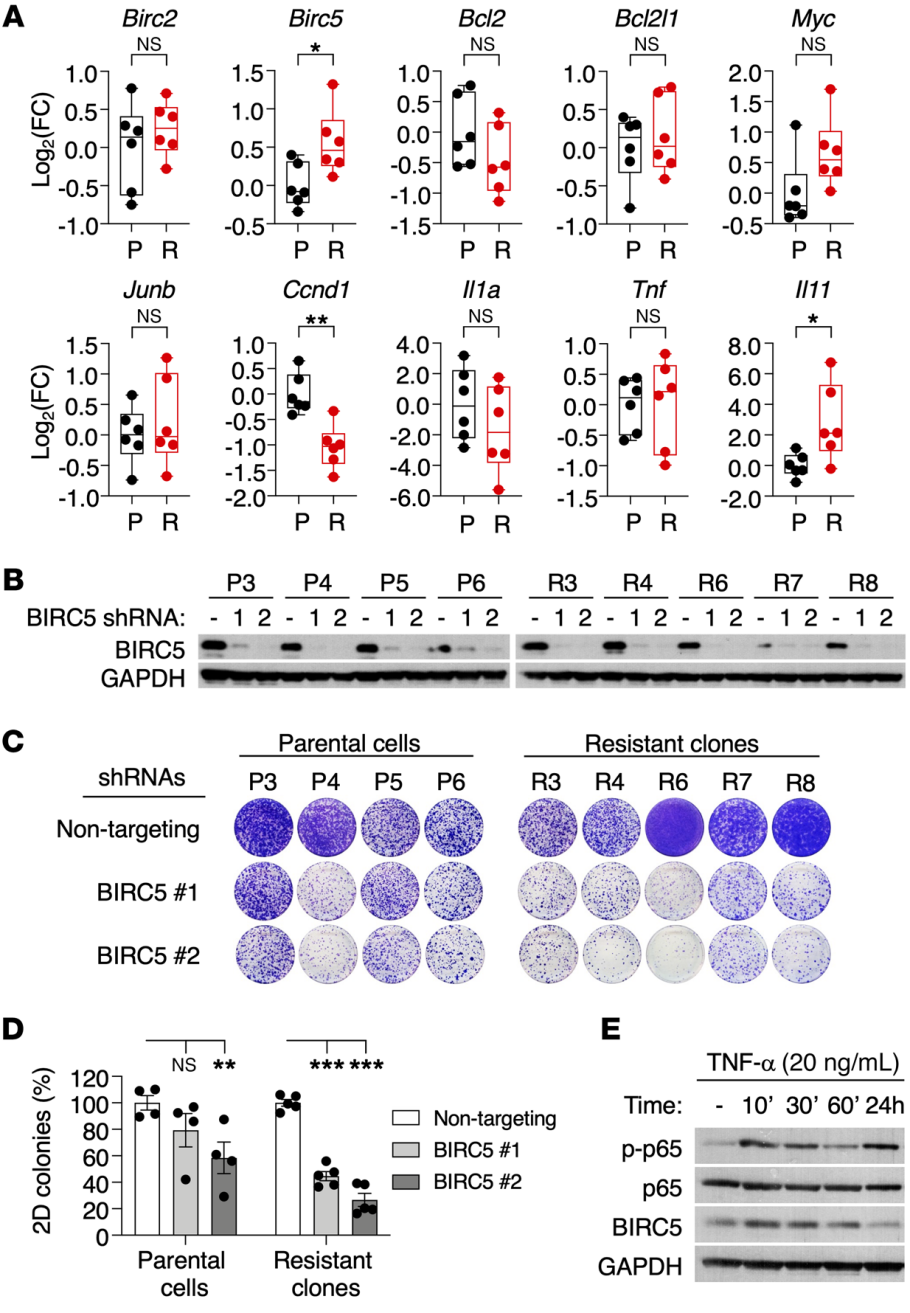
BIRC5 is required for survival of resistant *Kras^+/–^;Trp53^–/–^* clones. (**A**) Shown are log_2_ fold change (FC) values determined by quantitative reverse-transcriptase PCR (qRT-PCR) for NF-κB target genes in parental *Kras*^+/G12Vlox^;*Trp53^–/–^* cells (P, black, *n* = 6) and resistant *Kras^+/–^*;*Trp53^–/–^* clones (R, red, *n* = 6). β-Actin was used for normalization. *P* values were calculated using unpaired Student’s *t* test. *P < 0.05; **P < 0.01. (**B**) Western blot analysis of BIRC5 expression in lysates from parental *Kras*^+/G12Vlox^;*Trp53^–/–^* cell lines (P3 to P6) and resistant *Kras^+/–^*;*Trp53^–/–^* clones (R3 to R8) after expression of 2 independent shRNAs. GAPDH served as loading control. (**C**) Colony-formation assays on 10 cm cell culture dishes of parental *Kras*^+/G12Vlox^;*Trp53^–/–^* cell lines and resistant *Kras^+/–^*;*Trp53^–/–^* clones expressing either nontargeting shRNA or 2 independent shRNAs against BIRC5. (**D**) Quantification of the number of colonies present in the experiment described in **C**. Data are represented as mean ± SEM. *P* values were calculated using 2-way ANOVA. **P < 0.01; ***P < 0.001. (**E**) Western blot analysis of p-p65, p65, and BIRC5 expression in lysates from a parental *Kras*^+/G12Vlox^;*Trp53^–/–^* cell line treated with TNF-α (20 ng/ml) for the indicated time points. GAPDH served as loading control.

To determine whether activation of these pathways was mechanistically linked to the ability of these cells to proliferate upon *Kras*^G12V^ ablation, we used shRNAs to inhibit p65 and/or STAT3 expression (Supplemental Figure 7, A and B). As illustrated in Figure 4, B and C, inhibition of p65 expression effectively inhibited colony formation of the *Kras^+/–^*;*Trp53^–/–^* clones. Downregulation of STAT3 also blocked colony formation, but the inhibitory activity was less potent, especially considering its effect on the parental cell lines. Combined inhibition of both proteins further increased the inhibitory effect of p65 downregulation in the *Kras^+/–^*;*Trp53^–/–^* clones (Figure 4, B and C). Pharmacological interference with NF-κB signaling (using the NF-κB inhibitor BAY 11-7082) and/or STAT3 (using the STAT3 inhibitor Stattic) yielded similar results (Figure 4D). To identify the mechanism by which NF-κB/STAT3 signaling mediates resistance to *Kras*^G12V^ ablation, we determined the expression levels of several known NF-κB target genes in resistant *Kras^+/–^*;*Trp53^–/–^* clones compared with the parental cell lines. As illustrated in Figure 5A, we noted elevated expression of *Birc5* (also known as survivin), a common NF-κB/STAT3 target gene (24, 25). Silencing of BIRC5 expression more effectively blocked colony formation in these resistant clones, suggesting that BIRC5 upregulation mediates, at least to some extent, resistance to *Kras*^G12V^ ablation in vitro (Figure 5, B–D). Indeed, activation of NF-κB signaling via TNF-α treatment resulted in increased BIRC5 expression, confirming the link between NF-κB and BIRC5 (Figure 5E). These observations, taken together, illustrate that *Kras*^G12V^/*Trp53* mutant lung tumor cells can survive *Kras*^G12V^ ablation through activation of NF-κB– and STAT3-driven pathways. Whether these pathways also play a role in clinically resistant tumors remains to be determined.

### Pharmacological inhibition of oncogenic KRAS signaling induces rapid tumor resistance.

The development of selective KRAS^G12C^ inhibitors has allowed us to interrogate the efficacy of blocking oncogenic KRAS signaling in experimental lung tumors and eventually to study those mechanisms responsible for the development of resistance. To this end, we have generated a *Kras*^FSFG12C^ allele using homologous recombination in embryonic stem cells (Supplemental Figure 8, A and B). Intranasal infection of *Kras*^+/FSFG12C^;*Trp53*^F/F^ mice (designated as K^G12C^P) with Adeno-FLPo particles induced the development of LUADs indistinguishable from those previously observed in *Kras*^FSFG12V^;*Trp53*^F/F^ mice (26). These tumors appeared with full penetrance, although they displayed a slightly longer latency than those induced by the G12V mutation (Supplemental Figure 8C).

**Figure 6:**
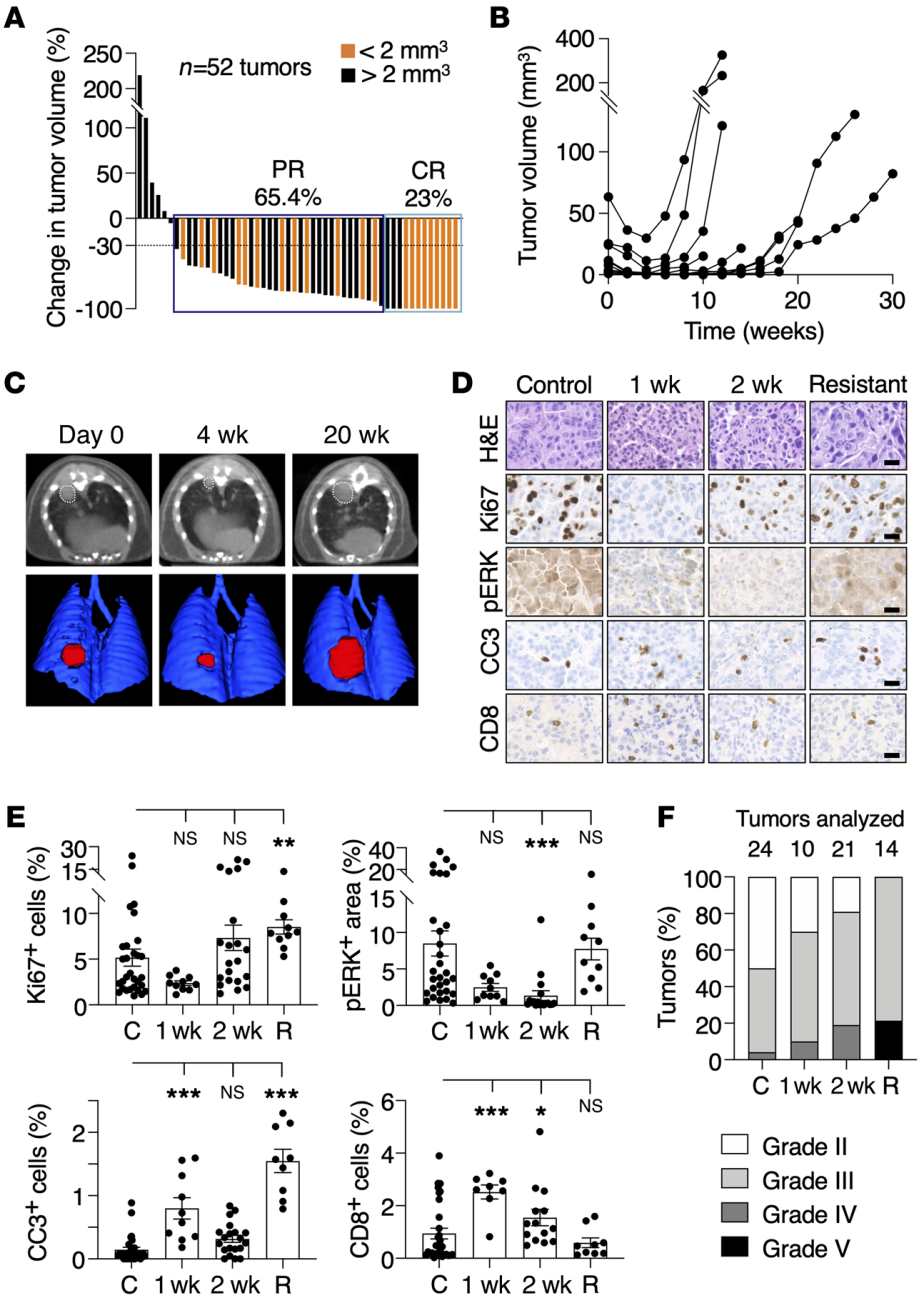
Tumor response to sotorasib treatment in K^G12C^P mice. (**A**) Waterfall plot representing the changes in tumor volumes of individual lung tumors present in *Kras*^+/FSFG12C^;*Trp53*^F/F^ (K^G12C^P) mice treated with sotorasib for 1 month (*n* = 15 mice/52 tumors). Tumors whose volume at the time of the first CT were smaller (orange) or larger (black bars) than 2 mm^3^ are indicated. (**B**) Tumor volumes determined by CT scans of representative tumors in K^G12C^P mice treated with sotorasib for the indicated times. (**C**) CT scans (top) and 3D rendering (bottom) of lungs of K^G12C^P mice at the beginning (day 0) and after sotorasib treatment for 4 and 20 weeks (w). Visible lesions are outlined by dotted lines (above) and in red (bottom). (**D**) Representative images of H&E, Ki67, pERK, cleaved caspase-3 (CC3), and CD8 staining in paraffin-embedded sections of tumors from K^G12C^P mice either untreated (Control), treated with sotorasib for 1 or 2 weeks, and after they became resistant to sotorasib (Resistant). Scale bars: 20 μm. (**E**) Quantification of the percentages of Ki67^+^, CC3^+^, and CD8^+^ cells and pERK^+^ areas in sections of tumors from K^G12C^P mice either untreated (C) or treated with sotorasib for 1 or 2 weeks, and after they became resistant to sotorasib (R). Data are represented as mean ± SEM. *P* values were calculated using 1-way ANOVA. *P < 0.05; **P < 0.01; ***P < 0.001. (**F**) Percentages of the different histological grades (II to V) displayed by lung tumors in K^G12C^P mice. Different shades of gray indicate increasing grades. Tumors present in K^G12C^P mice untreated, treated with sotorasib for 1 or 2 weeks, and after they became resistant to sotorasib are indicated.

K^G12C^P mice bearing 2 to 4 lung tumors that could be easily identified by CT scans were enrolled in a preclinical trial and treated with sotorasib (27–29). As illustrated in Figure 6A, continuous exposure of these tumor-bearing mice to the drug for 1 month at 100 mg/kg, a dose equivalent to that used in the CodeBreaK100 clinical trial (6), resulted in similar antitumor responses, with about two-thirds of the tumors displaying PRs and 23% CRs. Yet most of the CRs took place in tumors smaller than 2 mm^3^. Prolonged treatment of these mice with sotorasib caused the appearance of resistant tumors in all animals (*n* = 10) after 4 to 12 weeks of treatment (Figure 6, B and C). Acquisition of resistance appeared to be independent of the original size of the tumor, since those tumors that became resistant varied in size from 1.2 to 65 mm^3^ (Figure 6B).

As expected, treatment of established tumors in K^G12C^P mice with sotorasib caused a dramatic reduction in the Ki67 and pERK markers as well as an increase in the number of apoptotic cells (CC3^+^) accompanied by infiltration of CD8^+^ T cells (Figure 6, D and E). Resistant tumors did not differ from those in untreated controls, including restoration of Ki67 and pERK expression, except for the degree of apoptotic cells that remained elevated in the resistant tumors (Figure 6, D and E). Finally, resistant tumors displayed a clear trend toward higher histological grades (Figure 6F).

### Amplification of the Kras^G12C^ allele in sotorasib-resistant tumors.

**Figure 7:**
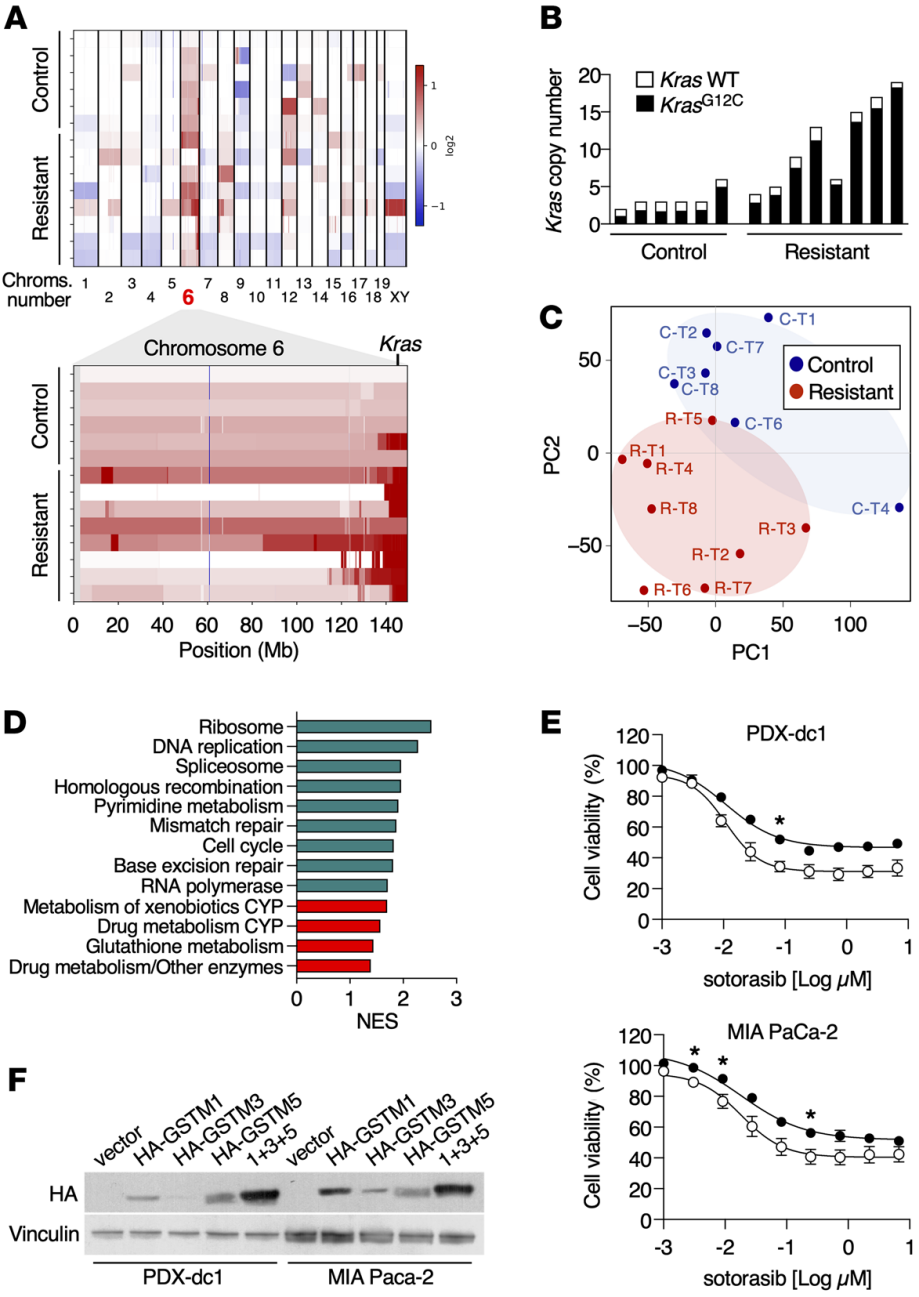
Genomic and transcriptomic analysis of sotorasib-resistant tumors. (**A**) Heatmaps representing log_2_ ratio copy number variations (CNVs) from WES data of control and sotorasib-resistant tumors in all chromosomes (top) and in chromosome 6 (bottom). Each row represents an individual sample. Copy number gains are represented in shades of red, while copy number losses are depicted in shades of blue. The position of *Kras* on chromosome 6 is indicated. (**B**) Absolute copy numbers of WT *Kras* (white bars) as well as *Kras*^G12C^ alleles (black bars) from control and sotorasib-resistant tumors. Data were obtained from WES analyses. (**C**) Principal component analysis (PCA) displaying the distribution of control tumors (blue) and sotorasib-resistant tumors (red). (**D**) Normalized enrichment scores of biological pathways significantly enriched in sotorasib-resistant tumors obtained from GSEA of KEGG gene sets. Proliferation-related pathways are represented in green and drug metabolism-related pathways in red. Only gene sets significantly enriched at FDR *q* values < 0.25 were considered. (**E**) Relative viability of PDX-dc1 and MIA PaCa-2 cells infected with empty lentiviral vectors or lentiviral vectors (white circles, *n* = 3 for MIA PaCa-2 cells, *n* = 2 for PDX-dc-1 cells)expressing GSTM1, GSTM3, and GSTM5 proteins (black circles, *n* = 3 for MIA PaCa-2 cells, *n* = 2 for PDX-dc-1 cells) after treatment with the indicated doses of sotorasib for 72 hours. *P* values were calculated using unpaired Student’s *t* test. *P < 0.05. (**F**) Western blot analysis of PDX-dc1 and MIA PaCa-2 cells infected with empty lentiviral vectors or lentiviral vectors expressing HA-GSTM1, HA-GSTM3, and/or HA-GSTM5 proteins using anti-HA antibodies. Vinculin expression served as a loading control.

To identify the mechanisms associated with resistance to sotorasib treatment, we submitted 8 resistant and 6 untreated control tumors to WES analysis. As depicted in Supplemental Table 2, we could not detect any de novo oncogenic mutations previously described in human tumors resistant to sotorasib or adagrasib (12–14). However, we noted robust amplifications of the genomic region of chromosome 6 encompassing the *Kras* locus in 7 out of 8 resistant tumors analyzed (Figure 7A, Supplemental Figure 9, and Supplemental Table 3), suggesting that amplification of the mutant *Kras*^G12C^ allele is a major driver of sotorasib resistance in this experimental model. Indeed, we also determined the absolute copy numbers of WT or mutant *Kras* alleles. This analysis revealed a strong allelic imbalance toward selective amplification of the mutant *Kras*^G12C^ allele (Figure 7B).

### Sotorasib-resistant tumors display elevated levels of xenobiotic metabolism pathways.

To further characterize these sotorasib-resistant tumors, they were submitted to RNA-Seq analysis (Supplemental Table 4). As illustrated in Figure 7C, resistant tumors clustered together and showed no overlapped distribution with the corresponding control tumors. Intriguingly, Gene Set Enrichment Analysis (GSEA) of differentially expressed genes revealed upregulation of gene sets involved in the metabolism of xenobiotics such as cytochrome P450 (CYP450) and glutathione-S-transferases (GSTs) (Figure 7D). These results suggest that altered sotorasib metabolism could, at least partially, contribute to tumor resistance due to a further reduction of its therapeutic efficacy. Ectopic expression of 3 of the most consistently upregulated genes involved in xenobiotic metabolism, *Gstm1*, *Gstm3*, and *Gstm5*, readily caused an increase in GST activity and resistance to sotorasib in PDX-dc1 and MIA Paca-2 human tumor cells known to be sensitive to sotorasib (30) (Figure 7, E and F, and Supplemental Figure 10), indicating that the GSTM class of detoxifying enzymes can modify the response to sotorasib, resulting in its reduced antitumor activity.

Of note, we did not observe a direct correlation between expression of these metabolic genes and oncogenic KRAS activity, suggesting that upregulation of these pathways was not an immediate consequence of KRAS^G12C^ inhibition in sotorasib-resistant tumors (Supplemental Figure 11). Moreover, we did not detect evidence for activation of NF-κB and/or STAT3-signaling pathways in sotorasib-resistant tumors (Supplemental Figure 12), indicating that resistance to sotorasib in vivo was primarily caused by mechanisms that reduce its effectiveness due to increased copy numbers of *Kras*^G12C^ and activation of xenobiotic metabolism pathways.

To determine whether these results could be translated to human tumors, we implanted pieces derived from a patient-derived *KRAS*^G12C^-positive xenograft (PDX) lung tumor in immunocompromised mice. Mice carrying a fragment of this PDX tumor in each flank were treated with vehicle or with 100 mg/kg sotorasib. As illustrated in Supplemental Figure 13A, these PDX tumors became resistant to the drug at about 150 days of treatment and were subsequently submitted to WES analysis. As shown in Supplemental Figure 13B, they displayed de novo mutations in genes known to be altered in LUADs, but the consequences of most of these mutations remain to be determined (Supplemental Table 5). However, we did not identify amplifications in *KRAS*, such as those described in the K^G12C^P mouse model, or additional mutations in the *KRAS* oncogene, as previously described in clinical samples (Supplemental Figure 13, B and C, and Supplemental Table 6) (12–14). Remarkably, RNA-Seq analysis also revealed upregulation of a pathway related to xenobiotic metabolism, suggesting that metabolic modification of sotorasib may also occur in human tumors, although the specific GST family members upregulated in resistant tumors differed from those identified in murine tumors (Supplemental Figure 13, D and E, and Supplemental Table 7).

### Sotorasib resistance is reversible.

**Figure 8:**
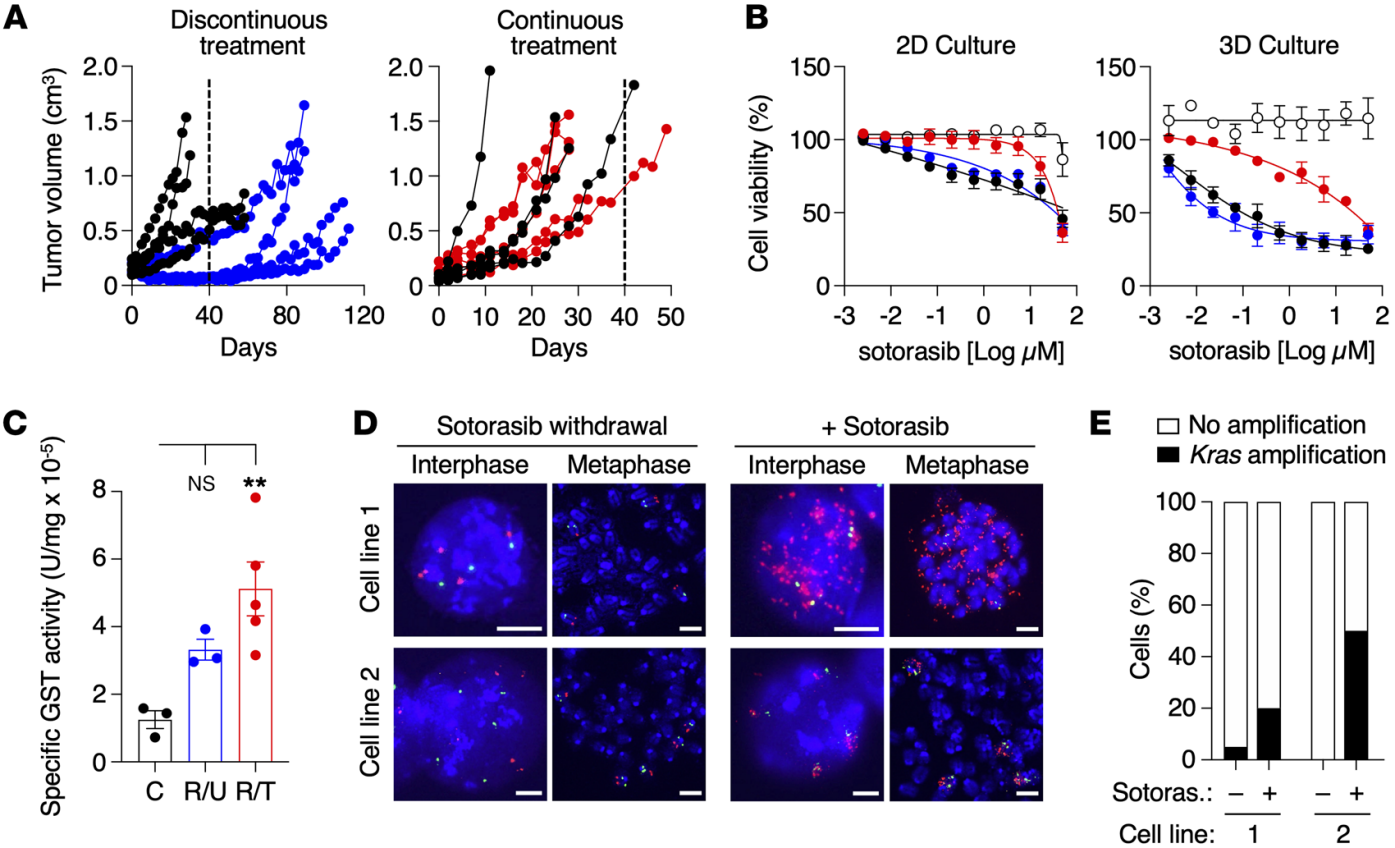
Resistance to sotorasib is reversible. (**A**) (Left) Growth of sotorasib-resistant tumors after subcutaneous implantation in immunodeficient mice treated either with vehicle (black circles) or 100 mg/kg sotorasib following a discontinuous treatment (blue circles) in which they were preexpanded in untreated immunodeficient mice. (Right) Growth of sotorasib-resistant tumors after subcutaneous implantation in immunodeficient mice treated either with vehicle (black circles) or with 100 mg/kg sotorasib following a continuous treatment (red circles) in which they were preexpanded in immunodeficient mice continuously treated with 100 mg/kg of sotorasib. Dotted lines indicate the differential time scale for tumors to become sotorasib resistant following discontinuous versus continuous exposure to sotorasib. (**B**) Viability of lung tumor cells treated with the indicated concentrations of sotorasib for 72 hours in 2D (left) and 3D (right) cultures. Tumor cells derived from untreated tumors expressing *Kras*^G12V^ (white circles, *n* = 3) or *Kras*^G12C^ (black circles, *n* = 3) as well as tumor cells obtained from sotorasib-resistant tumors either left untreated in culture (blue circles, *n* = 4) or cultured in the presence of 10 μM sotorasib (red circles, *n* = 2). Data are represented as mean ± SEM. (**C**) Specific GST activity (U/mg) of untreated control *Kras*^G12C^ tumor cells, tumor cells obtained from sotorasib-resistant tumors untreated in vitro (R/U), and tumor cells obtained from sotorasib-resistant tumors cultured in the presence of 10 μM of sotorasib (R/T). Colors are those described in **B**. Data are represented as mean ± SEM. *P* values were calculated using an ANOVA test. **P < 0.01. (**D**) Representative images of interphase and metaphase FISH analyses of 2 cell lines (1 and 2) obtained from sotorasib-resistant tumors, cultured in the absence of sotorasib (sotorasib withdrawal) or in the presence of 10 μM of sotorasib (+ sotorasib). Scale bars: 5 μm. (**E**) Absence (white bars) or presence of *Kras* (black bars) amplification in cell lines 1 and 2 grown in the absence (–) or presence (+) of 10 μM of sotorasib.

Resistant tumors expanded in immunocompromised mice under continuous exposure to sotorasib (100 mg/kg) rapidly resumed tumor growth when reimplanted in nude mice. In contrast, resistant tumors that were expanded in the absence of the drug and reimplanted in nude mice under similar experimental conditions took a significantly longer time to grow in the presence of the drug (Figure 8A). Reversibility upon drug withdrawal was also observed in vitro, since cell lines derived from resistant tumors became sensitive after sotorasib withdrawal, whereas those maintained under continuous exposure to sotorasib in vitro exhibited higher levels of resistance (Figure 8B). Finally, in agreement with previous observations, sotorasib inhibition was more effective in 3D spheroid cultures than in 2D monolayer cultures (31). This phenomenon was accompanied by a modulation of the GST activity levels, which remained elevated in cultured cells in the presence of sotorasib while decreasing upon drug withdrawal (Figure 8C). Fluorescence in situ hybridization (FISH) analysis on metaphase chromosomes revealed *Kras* amplifications on extrachromosomal DNA (ecDNA, also known as double-minutes) in some sotorasib-resistant cell lines maintained under continuous treatment, whereas others exhibited intrachromosomal amplifications (homogeneously staining regions, HSRs) (Figure 8D). Interestingly, when these cell lines were maintained in the absence of the drug, they no longer displayed amplifications of the *Kras* locus (Figure 8D). We also noticed a high degree of heterogeneity within the cell cultures, observing amplifications in only a percentage of cells (Figure 8E).

## Discussion

KRAS oncoproteins have long been considered undruggable targets, limiting the therapeutic options of cancer patients with *KRAS* mutations to standard cytotoxic treatments. This scenario has now changed with the development of selective KRAS^G12C^ and KRAS^G12D^ inhibitors, which target some of the most frequent *KRAS* mutations (10, 11, 16). Unfortunately, patients treated with KRAS^G12C^ inhibitors develop resistance within a few months, hence limiting the therapeutic value of these new drugs (12–14).

In this study, we provide genetic evidence that complete elimination of *Kras* oncogene expression in advanced *Kras*^G12V^;*Trp53^–/–^* LUADs completely prevented not only tumor progression, but also tumor maintenance. Indeed, most tumor cells disappeared in a relatively short period of time, primarily due to a dramatic reduction in cell proliferation and abundant apoptosis. We also detected a dramatic reduction in activation of the MAPK pathway (16, 32). Perhaps most importantly, none of the tumor cells became resistant in the absence of *Kras* oncogene expression, with a single exception. Indeed, out of more than 150 tumors, we only observed one resistant tumor due to the presence of a Q61H de novo mutation in the normal *Kras* allele. This observation, albeit limited to a single case, is reminiscent of those observed in human tumors in which resistance appears to be due to the presence of de novo mutations in either the *KRAS*^G12C^ oncogene or in loci involved in KRAS signaling (12–15). Yet about half of the human resistant tumors did not carry additional de novo mutations (13, 14). Therefore, our results indicate that one of the main drivers of tumor resistance might be a direct consequence of the limited blockade of KRAS signaling by current inhibitors. To what extent KRAS signaling needs to be inhibited to prevent the appearance of resistant cells is a critical issue, the resolution of which is needed to evaluate future pharmacological strategies in the clinic.

Ablation of *Kras* expression in tumors induced by urethane, a chemical carcinogen known to induce lung tumors with *Kras* mutations (21, 33, 34), also led to either complete or significant (>80%) regression of all tumors. In this model, we did not observe resistant tumors, possibly because both *Kras* alleles were eliminated upon TMX exposure. These results indicate that the requirement for *Kras* oncogene expression for tumor maintenance and progression is independent of the activating mutation, G12V in the genetic model and Q61R/L in the urethane model.

Regardless of the dramatic results observed in vivo, lung tumor cells can survive in the absence of *Kras* oncogene expression, as demonstrated by ablation of the resident *Kras* oncogene present in cell lines derived from *Kras*^G12V^;*Trp53* mutant LUADs. Resistant cells displayed activation of NF-κB and, to a lesser extent, STAT3 signaling, two pathways that frequently cooperate to promote the development and progression of certain tumors (35). Moreover, NF-κB and/or STAT3 activation have repeatedly been linked to resistance to targeted therapies, especially in the case of EGFR inhibitors (36–38). Our results show that proliferating *Kras* oncogene–deprived cells strongly depend on the combined activity of these transcription factors. Indeed, concomitant targeting of both pathways severely affected resistant tumor cells, but not those tumor cells that retained *Kras*^G12V^ expression. We also identified *Birc5* (survivin) as a common target gene of NF-κB and STAT3, mediating, at least to some extent, resistance to *Kras*^G12V^ ablation. BIRC5 is generally considered to be involved in antiapoptotic signaling and has also been linked to resistance (39, 40). Whether increased NF-κB/STAT3 signaling or BIRC5 expression may drive resistance to KRAS inhibition in the clinic remains to be determined.

*Kras*^G12C^;*Trp53*-driven mouse LUADs respond to sotorasib in a manner highly reminiscent of the clinical results described in the CodeBreaK100 phase I/II clinical trial (6, 27), indicating that this GEM tumor model effectively recapitulates the response of human tumors to pharmacological KRAS inhibition (6). Moreover, all *Kras*^G12C^;*Trp53* mice developed sotorasib resistance with proportionally similar kinetics to those observed in the clinic, keeping in mind the differential life spans of mice and humans. As indicated above, we did not detect those mutations present in other driver genes identified in circulating DNA of human patients, possibly due to the limited mutational burden of these tumors, in which secondary mutations are infrequent (12–14). Yet we detected both intra- (HSR) and extrachromosomal amplifications (ecDNA, also known as double minutes) of *Kras*^G12C^ as well as upregulation of gene-expression programs related to xenobiotic metabolism, suggesting that these tumors became unresponsive to sotorasib due to an imbalance between the amount of KRAS^G12C^ protein and the availability of the drug. Interestingly, amplifications of *KRAS* were also detected in 2 out of 10 LUAD patients who acquired resistance to adagrasib (13). Moreover, 3 additional LUAD patients had mutations in *KRAS* that prevented proper sotorasib binding, a conceptually similar mechanism (13). Additional mechanisms, such as expression of the efflux transporter ABCB1 or reduction of plasma availability, have been shown to limit sotorasib efficacy (41).

Our results revealed that distinct GSTM family members were consistently upregulated in resistant tumors as well as in cell lines and contributed to reduced inhibitory activity of sotorasib. Interestingly, a human PDX tumor that acquired resistance to sotorasib also displayed activation of a gene-expression program related to xenobiotic metabolism, although the individual GST family members upregulated were different from those found in resistant tumors in mice. Moreover, increased activation of xenobiotic metabolism was also recently reported in sotorasib-resistant lesions from a patient autopsy (42). Future studies are expected to reveal the precise mechanism of how these enzymes promote sotorasib resistance.

We also observed that tumor cells could adapt to the presence of the drug by maintaining a higher proportion of cells with amplifications of *Kras*^G12C^ in HSRs and ecDNA as well as elevated GST activity. When the tumors were no longer challenged with sotorasib, these traits disappeared. Similar observations have been made in colorectal cancer patients who acquired resistance to KRAS^G12C^ inhibitors via recurrent *KRAS*^G12C^ amplifications (43) or in lung cancer cells that acquired resistance to gefitinib carrying the *EGFR*^T790M^ oncogene within ecDNA (44). Moreover, extrachromosomal amplifications in ecDNA were lost upon drug withdrawal, suggesting that they only provided a selective advantage in the presence of sotorasib. Similarly, cells harboring intrachromosomal amplifications also lost their growth advantage upon drug withdrawal, suggesting that these amplifications may have deleterious effects in the absence of the drug, a concept previously reported in colorectal cancer cell clones that became resistant to selumetinib (45, 46). Notably, adaptive *KRAS* amplifications due to a strong selective pressure to increase *KRAS* copy numbers during the treatment might be prevented by applying an intermittent treatment regimen rather than a continuous schedule (47, 48). Other mechanisms of adaptive resistance to KRAS^G12C^ inhibition, including feedback upregulation of a variety of RTKs or AURKA, have been described (14, 49–51). Overall, our study provides evidence that resistance to sotorasib may occur via adaptive mechanisms that limit the on-target activity of the drug.

Finally, we have also observed substantial remodeling of the tumor microenvironment under both experimental scenarios, drug inhibition and genetic ablation. It is known that KRAS contributes to an immune-suppressive microenvironment and, consistent with previous reports, both strategies caused a strong increase in CD8^+^ T cells (52, 53). Our data suggest that T cells are not necessary to halt tumor progression upon *Kras*^G12V^ elimination, but they contribute to achieving CRs. Similar results were reported in a recent study using an immunocompetent model of pancreatic ductal adenocarcinoma (PDAC) treated with the KRAS^G12D^ inhibitor MRTX1133 (11). Notwithstanding the improved therapeutic efficacy of forthcoming KRAS inhibitors, their combination with checkpoint inhibitors or with selective inhibitors of upstream (SOS1, SHP2, RTKs) and downstream (MAPK pathway) effectors is likely to improve the efficacy of anti-KRAS therapies in the clinic.

## Methods

### Mice.

The *Kras*^FSFG12V^ (26), *Trp53*^frt^ (*Trp53*^F^) (54), *Tg.hUBC-CreERT2*^T^ (55), and *Rosa26-CreERT2*^KI^ (56) strains have been described previously. Generation of *Kras*^FSFG12Vlox^ and *Kras*^FSFG12C^ alleles is described in Supplemental Figure 1 and Supplemental Figure 8, respectively. Animals were maintained in a mixed 129/Sv-C57BL/6 background. Female and male mice were used for the experiments. Immunodeficient NU-Foxn1^nu^ mice (females, 5 weeks old) were purchased from Harlan Laboratories. All mice were genotyped at the CNIO Genomics Unit.

### Cell lines.

Cell lines were generated from tumors established in untreated K^G12Vlox^P, K^G12Vlox^PC2, or K^G12C^P mice as well as in sotorasib-resistant K^G12C^P mice. MIA PaCa-2 cells were obtained from ATCC. PDX-dc1 cells have been described (26). All cells were grown in DMEM supplemented with 10% FBS.

### Histopathology and immunohistochemistry.

Tissues were fixed in 10% buffered formalin (MilliporeSigma) and embedded in paraffin. For histopathological visualization, 2.5 μm tissue sections were stained with H&E, and tumors were classified according to standard histopathological grade criteria (20). Antibodies used for immunostaining included those against the following: Ki67 (1:50, Cell Signaling Technology, 12202), phospho-ERK (1:300, Cell Signaling Technology, 9101), cleaved caspase-3 (1:300, Cell Signaling Technology, 9661), and CD8 (1:200, Monoclonal Antibodies Core Unit, CNIO, OTO94A). For imaging analysis, slides were scanned (Axio Scan.Z1, Zeiss) and processed using ZEISS ZEN, version 3.1, software.

### Western blot analysis.

Protein extraction was performed in protein lysis buffer (50 mM Tris-HCl pH 7.5, 150 mM NaCl, 0.5% NP-40) supplemented with a cocktail of protease and phosphatase inhibitors (cOmplete Mini, Roche; Phosphatase Inhibitor Cocktail 2 and 3, MilliporeSigma). A total of 30 μg of protein extracts was separated on NuPAGE 4%–12% Bis-Tris Midi Gels (Invitrogen), transferred to a nitrocellulose blotting membrane (GE Healthcare), and blotted with antibodies against the following: EGFR (Abcam, ab52894), phospho-EGFR (Abcam, ab40815), ERK1 (BD Biosciences — Pharmingen, 554100), ERK2 (BD Biosciences, 610103), phospho-ERK1/2 (Cell Signaling Technology, 9101), AKT (Cell Signaling Technology, 9272), phospho-AKT (Cell Signaling Technology, 9271), MEK1 (Santa Cruz Biotechnology Inc., sc-6250), MEK2 (BD Biosciences, 610235), phospho-MEK1/2 (Cell Signaling Technology, 9154), NF-κB p65 (Santa Cruz Biotechnology Inc., sc-372), phospho–NF-κB p65 (Cell Signaling Technology, 3031), STAT3 (Cell Signaling Technology, 9139), phospho-STAT3 (Cell Signaling Technology, 9131), caspase-3 (Cell Signaling Technology, 9662), cleaved caspase-3 (Cell Signaling Technology, 9661), pan-RAS (Calbiochem, OP40), BIRC5 (Cell Signaling Technology, 2808), Lamin B (Santa Cruz Biotechnology Inc., sc-6216), HA.11 (BioLegend, 901513), GAPDH (MilliporeSigma, G8795), and Vinculin (MilliporeSigma, V9131).

### Data availability.

RNA-Seq data have been deposited in the NCBI’s Gene Expression Omnibus database (GSE204752, resistant and control tumors from the GEM model; GSE204753, control and resistant human PDX samples; GSE204754, analysis of tumor-derived cell lines and resistant clones ). WES data were deposited in the NCBI’s Sequence Read Archive (SRA PRJNA839872, sequencing of resistant and control tumors in GEM models; PRJNA840932, sequencing of resistant and control human PDX samples).

### Statistics.

Data are represented as mean ± SEM. *P* values were calculated with unpaired, 2-tailed Student’s *t* test and 1-way or 2-way ANOVA tests, where indicated, using GraphPad Prism (version 8.4.0) software. *P* values of less than 0.05 were considered statistically significant.

### Study approval.

All animal experiments were approved by the Ethical Committees of the Spanish National Cancer Research Centre (CNIO), the Carlos III Health Institute, and the Autonomous Community of Madrid and were performed in accordance with the guidelines stated in the International Guiding Principles for Biomedical Research Involving Animals, developed by the Council for International Organizations of Medical Sciences (CIOMS). Mice were housed under specific pathogen–free conditions at CNIO’s Animal Facility (Association for Assessment and Accreditation of Laboratory Animal Care, JRS: dpR 001659).

For further information, see Supplemental Methods.

## Author contributions

M Salmón performed most of the experiments, analyzed and interpreted the data, and contributed to writing the paper. RAD, CFT, and FAS performed bioinformatics analyses. M Sanclemente and MM contributed to urethane injections, mouse treatments, and PDX experiments. OB validated differentially expressed genes. FFG and LMC participated in cell-culture experiments. ALG performed drug treatments in mice. MCMG and SRP performed FISH analyses. CGL established resistant clones. EBM performed Southern blots. FM was responsible for CT acquisitions and analyses. LM was involved in flow cytometry experiments. OD contributed to RNA-Seq and allelic comparisons. EC performed histopathological analyses. SO was responsible for embryonic stem cell electroporations and microinjections to establish GEM models. CG contributed critical information and generated *Kras^lox^* mice. MD and MB conceptualized the study, designed the experiments, interpreted the data, and wrote the paper.

## Supplementary Material

Supplemental data

Supplemental table 1

Supplemental table 2

Supplemental table 3

Supplemental table 4

Supplemental table 5

Supplemental table 6

Supplemental table 7

## Version 1 03/16/2023

In-Press Preview

## Version 2 04/03/2023

Electronic publication
