# Supplementary material for: Kras oncogene ablation prevents resistance in advanced lung adenocarcinomas
Source: J Clin Invest. 2023 Apr 3;133(7):e164413. doi: 10.1172/JCI164413 (PMC10065067; doi:10.1172/JCI164413)
Supplement: Supplemental data [file jci-133-164413-s191.pdf]

SUPPLEMENTAL MATERIALS FOR

Kras oncogene ablation prevents resistance in advanced lung adenocarcinomas

Marina Salmón,[1] Ruth Álvarez-Díaz,[1] Coral Fustero-Torre,[2] Oksana Brehey,[1] Carmen G. Lechuga,[1] Manuel Sanclemente,[1] Fernando Fernández-García,[1] Alejandra López-García,[1] María Carmen Martín-Guijarro,[3] Sandra Rodríguez-Perales,[3] Emily Bousquet-Mur,[1] Lucía Morales-Cacho,[1] Francisca Mulero,[4] Fátima Al-Shahrour,[2] Lola Martínez,[5] Orlando Domínguez,[6] Eduardo Caleiras,[7] Sagrario Ortega,[8] Carmen Guerra,[1,10] Monica Musteanu,[1,9,10] Matthias Drosten,[1,10,11,12,*], and Mariano Barbacid[1,10,12,*]

*Correspondence to: mdrosten@usal.es or mbarbacid@cnio.es.

This PDF file includes:

Supplemental Methods

Supplemental References

Supplemental Figures 1 to 13

SUPPLEMENTAL METHODS

Generation of *Kras*^{FSFG12Vlox} and *Kras*^{FSFG12C} mice. The targeting vector to generate the *Kras*^{FSFG12Vlox} allele was prepared by Gene Bridges GmbH. In brief, the genomic *Kras* locus was subcloned from BAC RP23-452H12 into a high-copy plasmid backbone via Red/ET recombination, incorporating AscI and PmeI restriction sites for convenient linearization prior to introduction into embryonic stem (ES) cells. The final targeting construct was generated by inserting the FRT-PGK-gb2-hygro STOP cassette and the floxed exon 1 carrying the G12V mutation into the high-copy plasmid harboring the *Kras* genomic locus by multi-site Red/ET cloning. All clones were analyzed by PCR to assess the correct modification of the plasmid. The linearized vector was electroporated into G4 ES cells. Recombinant ES cell clones were identified by Southern blot analysis. Two independent ES cell clones (ESMD26.13 and ESMD26.15) were microinjected into C57BL/6J blastocysts to generate chimeric mice. Male chimeras were crossed with C57BL/6J females to obtain germline transmission of the targeted allele.

The targeting vector to generate the *Kras*^{FSFG12C} allele was generated by modifying the *Kras*^{FSFG12V} targeting vector (1) by site-directed mutagenesis. Correct modification of the vector was assessed by Sanger sequencing analysis. The resulting targeting vector was linearized with NotI and electroporated into G4 ES cells. ES cell clones were submitted to Southern blot analysis to identify those clones undergoing the expected recombination event. Two ES cell clones (ESMD31.281 and ESMD32.144) were microinjected into C57BL/6J blastocysts. Male chimeras were crossed with C57BL/6J females to obtain germline transmission of the targeted allele.

Southern blot analysis. To identify recombinant ES cell clones carrying *Kras*^{FSFG12Vlox}, DNA was digested with NdeI (left arm) or SphI (right arm). Fragments were identified

with a 578 bp DNA probe corresponding to sequences located 3536 bp upstream of exon 0 (probe A), or with a 625 bp DNA probe corresponding to sequences located 4176 bp downstream of exon 1 (probe B), respectively. Enzymatic digestion resulted in the following bands: 12.7 kbp for the wild-type and 9.7 kbp for the recombinant alleles (probe A), or 7.4 kbp for the wild-type and 5.2 kbp for the recombinant alleles (probe B), respectively (Supplemental Figure 1).

To select recombinant ES cell clones carrying the *Kras*^{FSFG12C} allele, DNA was digested with NsiI and the resulting fragments were identified with a 451 bp DNA probe corresponding to sequences located 574 bp upstream of exon 0 (5' probe), or with a 498 bp DNA probe corresponding to sequences located 1696 bp downstream of exon 1 (3' probe), respectively. Enzymatic digestion resulted in the following diagnostic bands: 14.0 kbp for recombinant allele and 10.7 kbp for the wild-type allele, detected either with the 5' or the 3' probe (Supplemental Figure 8).

Tumor induction and tamoxifen exposure. Lung tumor formation was induced in anesthetized (100 mg/kg ketamine and 10 mg/kg xylazine) 8-10 weeks old mice via intranasal inoculation of 10^6 or $3x10^7$ pfu per mouse of adenoviral particles expressing the FLPo recombinase (Adeno-FLPo), which concomitantly eliminates the transcriptional termination cassette preceding the *Kras*^{G12C} or *Kras*^{G12Vlox} oncogenes, as well as *Trp53* alleles. Activation of the inducible CreERT2 recombinases encoded by the *hUBC-CreERT2* and *Rosa26-CreERT2* alleles was carried out by feeding the mice with a Tamoxifen (TMX)-containing diet (Teklad CRD TAM400 diet, Harlan) *ad libitum* during the duration of the experiment. The tumor response was monitored by CT analysis. Correct target ablation was assessed by PCR analysis, using the following specific primers for *Kras*^{G12Vlox} allele: forward primer (5'-

GGAACTTCGCATGCATAACTTCGTATAATGT-3’) and reverse primer (5’-CAAAGCACGGATGGCATCTTGGACC-3’), yielding a 594 bp band corresponding to the unexcised allele and a 170 bp band for excised allele.

In vivo pharmacological treatments. Drug treatments were carried out in tumor-bearing $K^{G12C}P$ mice. Sotorasib (MedChemExpress) was administrated on 5 consecutive days per week by oral gavage (100 mg/kg), dissolved in 2% hydroxypropyl methylcellulose (HPMC) and 1% Tween 80. The tumor response was monitored by CT analysis.

Orthotopic and subcutaneous implantation of lung tumor cell lines. Implantations were carried out in immunodeficient $Foxn1^{nu}$ mice. Subcutaneous tumor formation was induced by injecting 10^6 cells resuspended in 100 µl of sterile PBS:Matrigel (1:1) (Corning, 354234) in the flank of anesthetized (4% isoflurane in 100% oxygen at a rate of 0.5 l/min) mice. The tumor size was measured periodically using a caliper and calculated as (short axis × short axis × long axis)/2. For transpleural orthotopic cell injections, 10^5 cells in 10 µl of sterile PBS:Matrigel (1:1) were injected through the intercostal space into the lung of anesthetized (4% isoflurane in 100% oxygen at a rate of 0.5 l/min) mice.

Implantation of mouse and PDX tumors in immunodeficient mice. PDX lung tumors (1) and lung tumors from $K^{G12C}P$ mice were cut into small fragments (approx. 5 mm^3), embedded in Matrigel and introduced through a transversal incision in the flank of anesthetized (4% isoflurane in 100% oxygen at a rate of 0.5 l/min) mice. The tumor size

was measured periodically using a caliper and calculated as (short axis × short axis × long axis)/2.

Micro-CT Imaging. Mice were anesthetized by inhalation of 4% isoflurane in 100% oxygen at a rate of 0.5 l/min. The parameters for CT acquisition were set as follows: 100 µA, 50 kV, projections 360, 10 shots, set binning 2x2 and set exposure 100 milliseconds. Lung images were acquired using a SuperArgus COMPACT (Sedecal). Image processing, analysis and 3D rendering was performed using the 3D Slicer Viewer Software. Tumor volume was calculated according to the following formula: (Short axis × short axis × long axis)/2.

Flow cytometry analysis. Tumors were dissected from lungs and single cell suspensions were obtained by mechanical dissociation, followed by enzymatic digestion with Hank´s Balanced Salt Solution (HBSS) (Gibco) + 1 mg/ml Collagenase IV (Gibco) + 0.1 mg/ml DNase I (Sigma). Erythrocytes were lysed with ACK lysis buffer (Lonza) and cells were resuspended in PBS containing 0.1% BSA and 2 mM EDTA. Then, cells were incubated with purified anti-mouse CD16/32 antibody (1 µl/10^6 cells) (BD Pharmingen) for 15 min at RT to block Fc binding. Aliquots of 10^6 cells were stained for 30 min on ice with the following monoclonal antibodies: FITC anti-CD45 (1:400, clone 30-F11 BD Pharmingen), PE anti-CD49b/Pan-NK Cells (1:200, clone DX5 BD Pharmingen), APC anti-CD3 (1:200, clone 145-2C11 BD Pharmingen), PerCP-Cy5.5 anti-CD4 (1:400, clone RM4-5 BD Pharmingen) and PE-Cy7 anti-CD8 (1:400, clone 53-6.7 BD Pharmingen). APC anti-F4/80 (1:400, clone BM8 eBioscience) and PE-Cy7 anti-CD11b (1:800, clone M1/70 BD Pharmingen) were included in a second multicolor panel for macrophage identification. 0.1 µg/ml of DAPI (Molecular Probes) was used to identify dead cells.

Samples were collected on a FACS Canto II flow cytometer (BD Biosciences) equipped with 488nm, 405nm and 633nm laser lines. Cell aggregates were excluded using pulse processing and at least 10,000 events of interest were collected. Data were analyzed using FlowJo V10 (BD Biosciences).

Cell proliferation and colony formation assays. 300 cells per well were dispensed in 96-well plates in DMEM supplemented with 10% FBS in triplicate, and proliferation was assessed by the MTT cell viability assay (Sigma) every two days. Cell growth was calculated relative to day 0. For 2D colony formation, 5000 cells per plate were cultured in 10 cm diameter dishes in DMEM supplemented with 10% FBS for 7-10 days. Then, plates were fixed with 0.1% glutaraldehyde and stained with 0.2% Crystal Violet. Colonies were counted and quantified. For 3D sphere formation, 1000 cells per well were plated in 96-well plates in equal parts of DMEM supplemented with 10% FBS and Matrigel (Corning, 354234) in triplicate, and incubated for 7 days. To visualize and quantify colony formation, images were captured using a Leica DMI6000 B microscope and LAS AF 2.7 software (Leica-Microsystems). Z-series projection for three-dimensional (3-D) reconstruction was performed using ImageJ (National Institutes of Health). To interrogate the effect of *Kras*^{G12V} ablation on proliferation, *Kras*$^{+/G12Vlox}$;*Trp53*$^{-/-}$;*Rosa26-CreERT2*$^{KI/KI}$ tumor cells were exposed to 600 nM 4-hydroxytamoxifen (4-OHT) (Sigma, H7904), harvested at 0, 8, 24, 48, 72 and 96h after exposure to 4-OHT and subjected to western blot analysis.

In vitro drug treatments. Cells (4000 cells per well) were dispensed in 96-well plates in DMEM supplemented with 10% FBS and grown for 24h. Alternatively, cell lines were seeded on ultra-low attachment 96-well plates, with clear round bottom and black opaque

microplate body (Corning, 4515) at a density of 4000 cells per well and grown for 4 days until spheroids formation. Then cells were treated with threefold dilutions of sotorasib (MedChemExpress), or with BAY 11-7082 (10 μM) and Stattic (5 μM) as single or combined treatments for 72h, and viability was determined by MTT or Cell Titer Glo assay (Promega). Every condition was seeded in triplicate and normalized to DMSO-treated controls. To calculate the IC_{50}, values were plotted and fit to a sigmoid dose–response curve using GraphPad Prism (v8.4.0) software. To activate NF-kB, cells were exposed to 20 mg/ml tumor necrosis factor α (TNF-α, R&D Systems).

Adenoviral infection. To obtain resistant tumor cells growing in the absence of *Kras*^{G12V}, cells (*Kras*$^{+/G12Vlox}$; *Trp53*$^{-/-}$) were seeded at a density of $1x10^3$ cells per plate and infected with Adeno-Cre-GFP (adenoviral particles expressing Cre recombinase and green fluorescent protein) at an MOI of 10 (multiplicity of infection). Then, individual colonies were harvested using sterile cloning cylinders (Sigma) and seeded on a 96-well plate for expansion.

Knockdown assays. Tumor cells were infected with lentiviral supernatants expressing shRNA against p65 (TRCN0000055346 and TRCN0000235832, Sigma), STAT3 (TRCN0000071453 and TRCN0000071454) and BIRC5 (TRCN0000335351 and TRCN0000054613). The TRCN0000071454 shRNA was cloned into a plasmid that carries a blasticidin resistance cassette for combined knockdowns. A non-targeting shRNA vector (SHC002) was used as negative control.

Cloning of lentiviral expression vectors. Lentiviral vectors expressing mouse GSTM1, GSTM3 and GSTM5 were generated by amplifying the corresponding cDNAs from pCMV-SPORT6-Gstm1, pCMV-SPORT6-Gstm3 and pCMV-SPORT6-Gstm5 (obtained from the CNIO Genomics Unit), respectively, by PCR. A hemagglutinin (HA) tag sequence was added to the 3' terminus within the PCR primers. PCR products were cloned into pCR8/GW/TOPO using the pCR8/GW/TPOP TA Cloning Kit (ThermoFisher Scientific), and, after sequence verification, transferred into the Gateway destination vectors pLentiCMVPuroDEST (Addgene 17452), pLentiCMVBlastDEST (Addgene 17451) and pLentiCMVHygroDEST (Addgene 17454), respectively, using the Gateway LR Clonase II Enzyme mix (ThermoFisher Scientific).

RAS activation assay. RAS-GTP levels were determined using the Ras Activation Assay kit (Cell Biolabs) according to manufacturer's instructions. Cell lysates were incubated with Raf-1 RBD (Ras Binding Domain) agarose beads for 1h at 4ºC in gentle agitation. After washing with lysis buffer, the beads were denatured in 1X loading buffer and 1X reducing agent (NuPAGE, Invitrogen), and boiled for 5 min at 95ºC. Pull-down samples and whole-cell lysates were detected by Western blot using anti pan-RAS antibodies.

Fluorescence in situ hybridization (FISH). Two sets of bacterial artificial chromosome (BAC) clones (RP24-359O2 and RP24-175B6 for *Kras* at 6qG3; and RP24-345H19 and RP24-275J18 for control probe at 6qA3.3) were obtained from the BACPAC Resources Centre (https://bacpacresources.org/) to generate a FISH probe to detect *Kras* amplification or chromosome 6 aneuploidies. The *Kras* probe was labeled with Spectrum-Orange and the control probe with Spectrum-Green. FISH analyses were performed as previously published (2). Briefly, cells exposed to colcemid to arrest mitosis at the

metaphase stage were treated with a hypotonic solution and fixed with glacial acetic acid and methanol. After dehydration, the samples were denatured in the presence of the specific probe at 73°C and incubated overnight for hybridization at 37°C. Finally, the slides were washed in 20X SSC buffer with Tween 20 and mounted on fluorescent mounting media (DAPI in antifade solution). A Leica DM 5500B fluorescence microscope with a 100x oil-immersion objective, Leica DM DAPI, green, and orange fluorescence filter cubes, and a CCD camera (Photometrics SenSys camera) connected to a PC running the Zytovision image analysis system (Applied Imaging Ltd., UK) with Z stack software was used to image 200 cells. The z-stack images were manually scored by two independent investigators by counting the number of co-localized signals.

GST activity assay. GST activity was detected with the colorimetric GST Assay kit (PromoCell) following manufacturer's recommendations. A total of 50 µg of protein lysate was loaded in 96-well plates in duplicate and the reaction was initiated by adding glutathione (GSH) and CDNB (1-Chloro-2,4-dinitrobenzene). The absorbance was measured every minute at 340 nm and the specific GST activity (U/mg) was calculated according to the manufacturer's guidelines.

qRT-PCR. Total RNA was extracted with the RNeasy Mini Kit (Qiagen) and reverse-transcribed using the SuperScript II Reverse Transcriptase kit (Invitrogen), random primers (Invitrogen) and dNTPs (ThermoFisher Scientific) following the manufacturer's instructions. Quantitative real time PCR reactions were performed on a QuantStudio 6 Flex Real-Time PCR System (Applied Biosystems) using the SYBR Green PCR Master

Mix (Applied Biosystems) with the primer pairs indicated below. β-actin was used for normalization.

Birc2: 5'-AGACTCTGCTTTCAGCCAGT-3' (forward) and 5'-AGAGCACAGGTTGGAGTGAA-3' (reverse)

Birc5: 5'-CTACCGAGAACGAGCCTGAT-3' (forward) and 5'-TGCTCCTCTATCGGGTTGTC-3' (reverse)

Bcl2: 5'-TCTGGTTGGGATTCCTACGG-3' (forward) and 5'-AGGAGGGTTTCCAGATTGGG-3' (reverse)

Bcl2l1: 5'-GACCCAGTAAGTGAGCAGGT-3' (forward) and 5'-GGAGAGAAAGTCGACCACCA-3' (reverse)

Myc: 5'-AGCGACTCTGAAGAAGAGCA-3' (forward) and 5'-CGTAGTTGTGCTGGTGAGTG-3' (reverse)

Junb: 5'-TCTACACCAACCTCAGCAGT-3' (forward) and 5'-ATGTGGGAGGTAGCTGATGG-3' (reverse)

Ccnd1: 5'-GAACTCTGTTCTCGCACCAC-3' (forward) and 5'-CCCAATCTCCTTGTCCAGGT-3' (reverse)

Il1a: 5'-AGTCAACTCATTGGCGCTTG-3' (forward) and 5'-GAGAGATGGTCAATGGCAGA-3' (reverse)

Tnf: 5'-CTCATGCACCACCATCAAGG-3' (forward) and 5'-ACCTGACCACTCTCCCTTTG-3' (reverse)

Il11: 5'-GGGATCTTTGCAGCTTCCTG-3' (forward) and 5'-TTGTACATGCCGGAGGTAGG-3' (reverse)

Gstm1: 5’-GGAGAACCAGGTCATGGACA-3’ (forward) and 5’-GGAACAGCCACAAAGTCAGG-3’ (reverse)

Gstm3: 5’-AGCCAGTCTGAGAAGACCAC-3’ (forward) and 5-GTGTATTCCAGGAGCAAGCG-3 (reverse)

Gstm4: 5’-GGGAGACGCTCCTGACTATG-3’ (forward) and 5’-CCACGCGAATCTTCTCTTCC-3’ (reverse)

Gstm5: 5’-TCCTAACCTGCCCTACCTCA-3’ (forward) and 5’-ATTGTAGCAGAGGCGAACCA-3’ (reverse)

Cyp2c23: 5’-ACCCTCGGGATTACATCGAC-3’ (forward) and 5’-AACTTTGGCTTGCACCTCTG-3’ (reverse)

Cyp2s1: 5’-TGGACGAAGATGGTCGGTTG-3’ (forward) and 5’-AAGAAAAGCCACAACTCCGC-3’ (reverse)

β-actin: 5’-GACGGCCAGGTCATCACTATTG-3’ (forward) and 5-AGGAAGGCTGGAAAAGAGCC-3 (reverse)

Subcellular fractionation. Nuclear and cytoplasmic fractions were extracted with the NE-PER Nuclear and Cytoplasmic Extraction Reagents (ThermoFisher Scientific) following manufacturer's protocols. Efficient nuclear and cytoplasmic fractionation was confirmed by western blot analysis using Lamin B antibodies for the nuclear fraction and GAPDH antibodies for the cytoplasmic fraction.

Whole exome sequencing. Genomic DNA was isolated from tumors using the salting-out method. Then, DNA samples were subjected to quality control and subsequent library

construction for whole exome sequencing (WES) using the SureSelect XT Mouse All Exon or SureSelect Human All Exon V6 libraries (Agilent Technologies) according to the manufacturer's recommendations. Paired-end Illumina sequencing (150 bp x bp) of the libraries was performed by Macrogen on a NovaSeq6000. The samples were sequenced to depths of approximately 18 Gb/sample.

For the alignment, quality control and detection of single nucleotide variants (SNVs) and small insertions and deletions (indels) a Snakemake pipeline (https://gitlab.com/bu_cnio/varca) based on the GATK best-practices workflow (3) was used. Briefly, Fastq format 150 bp paired-end reads were pre-processed using Cutadapt (4) to remove Illumina adapters. PDX samples underwent classification with Xenome (5) to discard contaminating reads from mouse stroma. Good quality reads were then aligned to the *Mus musculus* (GRCm38/mm10) or human (GRCh38) genome reference using the Burrows-Wheeler Aligner (BWA-MEM) (6). Following the alignment, we used Picard to mark duplicates and Genome Analysis Toolkit (GATK) for base quality score recalibration. Collection of alignment and coverage metrics was performed with samtools and Picard. Targeted bases were sequenced to a mean depth of 100x, with at least 80% of targeted bases sequenced to 30x coverage or higher.

Germline and medium-to-high frequency variants were detected using the GATK algorithm HaplotypeCaller, while MuTect2 was used for the calling of somatic low-frequency variants. Variants were selected using the hard-filtering recommendations from GATK, and annotated with SnpEff and Ensembl Variant Effect Predictor (VEP) tools (7, 8). Those variants reported as single nucleotide polymorphisms (SNPs) by the NCBI database dbSNP were removed. When population allelic frequencies were available (human), we also considered as polymorphisms those with allelic frequencies higher than 0.01. In mice, we generated a list of likely germline events by using a pooled

sample of tail DNA from two mice of mixed (129/Sv-C57BL/6) background and subtracted them from the list of variants detected in tumor samples.

Copy number variations (CNVs) were detected by using CNVkit software (9). A pooled copy-number reference was constructed from two control samples (DNA from mouse tails). Then, each sample was individually compared with the reference to calculate copy number ratios, and circular binary segmentation (CBS) algorithm (10) was used to infer copy number segments. Copy number variants were labelled as losses or gains relative to the overall sample wide estimated ploidy. Genes with copy number gains or losses above a threshold of 2 (by default), equivalent to single-copy gains and losses in a completely pure tumor sample, were reported. Also, CNVs covering <3 bins were not considered. Genome-wide/chromosome bin-level log2 coverages and segmentation calls for each sample were represented as a scatter plots, and a heatmap was drawn from copy number segments across all samples.

RNA sequencing and data analysis. Library construction was performed with either the TruSeq Stranded mRNA Sample Prep Kit (Illumina) or the QuantSeq 3' mRNA-Seq Library Prep Kit (Lexogen), and subsequently sequenced on an Illumina instrument following the manufacturer's protocols. PDX samples were pre-processed with Xenome to remove reads aligning to the mouse genome. Differentially expressed genes (DEG) were obtained using the Nextpresso pipeline (11). Sequencing quality was analyzed with FastQC (http://www.bioinformatics.babraham.ac.uk/projects/fastqc/); reads were aligned to the mouse (GRCm38/mm10) or human (GRCh38) genome using TopHat-2.0.10 (12), Bowtie 1.0.0 (13) and Samtools 0.1.19.0 (14); and transcript assembly, abundance estimation and differential expression were calculated with Cufflinks 2.2.1 (15). Where needed, the batch effect was adjusted using ComBat-seq (16). The estimated significance

level (*P* value) was corrected to account for multiple hypotheses testing using Benjamini and Hochberg False Discovery Rate (FDR) adjustment. Genes with FDR less than or equal to 0.05 were selected as differentially expressed. Gene Set Enrichment Analysis (GSEA) of differentially expressed genes was performed using the GSEA software, Version 2.0.6, obtained from the Broad Institute (17). GSEA was performed to analyze the enrichment of the gene sets following the developer's protocol and using pathway annotations from Gene Ontology, Reactome, Biocarta and Kyoto Encyclopaedia of Genes and Genomes (KEGG) databases. Differentially expressed genes were ranked according to their t-statistics. This ranked file was used as input for the enrichment analysis. All basic and advanced fields were set to default and only those gene sets significantly enriched at a False Discovery Rate (FDR) q-values < 0.25 where considered. A selection of significantly enriched gene sets was re-analyzed by using a single-sample GSEA (ssGSEA) with the R package "GSVA" (18). As a result, the sample-based normalized enrichment scores (NES) were obtained in order to reveal the underlying differences between samples. Heat-maps were generated using the software Morpheus.

Mass spectrometry analysis. Cells were lysed in protein lysis buffer (50 mM Tris-HCl pH 7.5, 150 mM NaCl, 0.5% NP-40) supplemented with a cocktail of protease and phosphatase inhibitors (cOmplete Mini, Roche; Phosphatase Inhibitor Cocktail 2 and 3, Sigma). A total of 120 µg of protein extract was loaded on NuPAGE 4-12% Bis-Tris gels (Invitrogen). Gels were stained with Coomassie Brilliant Blue R-250 staining solution (Bio-Rad) and a 0.5 cm^2 band corresponding to the estimated molecular weight of RAS proteins was excised. Mass spectrometric analysis of peptides unique for KRAS, $KRAS^{G12V}$, NRAS, HRAS, and peptides common to all RAS proteins were detected as previously described (19).

SUPPLEMENTAL REFERENCES

1. Sanclemente M, et al. c-RAF ablation induces regression of advanced Kras/Trp53 mutant lung adenocarcinomas by a mechanism independent of MAPK signaling. *Cancer Cell*. 2018;33(2):217-228 e4.

2. Rodriguez-Perales S, et al. Truncated RUNX1 protein generated by a novel t(1;21)(p32;q22) chromosomal translocation impairs the proliferation and differentiation of human hematopoietic progenitors. *Oncogene*. 2016;35(1):125-34.

3. McKenna A, et al. The Genome Analysis Toolkit: a MapReduce framework for analyzing next-generation DNA sequencing data. *Genome Res*. 2010;20(9):1297-303.

4. Martin M. Cutadapt removes adapter sequences from high-throughput sequencing reads. *EMBnetJournal*. 2011;17(1):10.

5. Conway T, et al. Xenome--a tool for classifying reads from xenograft samples. *Bioinformatics*. 2012;28(12):i172-8.

6. Li H. Aligning sequence reads, clone sequences and assembly contigs with BWA-MEM. arXiv: 1303.3997. 2013;

7. Cingolani P, et al. A program for annotating and predicting the effects of single nucleotide polymorphisms, SnpEff: SNPs in the genome of Drosophila melanogaster strain w1118; iso-2; iso-3. *Fly (Austin)*. 2012;6(2):80-92.

8. McLaren W, et al. The Ensembl Variant Effect Predictor. *Genome Biol*. 2016;17(1):122.

9. Talevich E, et al. CNVkit: Genome-wide copy number detection and visualization from targeted DNA sequencing. *PLoS Comput Biol*. 2016;12(4):e1004873.

10. Venkatraman ES, Olshen AB. A faster circular binary segmentation algorithm for the analysis of array CGH data. *Bioinformatics*. 2007;23(6):657-63.

11. Graña O, et al. Nextpresso: Next generation sequencing expression analysis pipeline. *Current Bioinformatics*. 2018;13(6):583-591.

12. Kim D, et al. TopHat2: accurate alignment of transcriptomes in the presence of insertions, deletions and gene fusions. *Genome Biology*. 2013;14(4):R36.

13. Langmead B, Salzberg SL. Fast gapped-read alignment with Bowtie 2. *Nature Methods*. 2012;9(4):357-359.

14. Li H, et al. The sequence alignment/Map format and SAMtools. *Bioinformatics*. 2009;25(16):2078-2079.

15. Trapnell C, et al. Transcript assembly and quantification by RNA-Seq reveals unannotated transcripts and isoform switching during cell differentiation. *Nature Biotechnology*. 2010;28(5):511-515.

16. Zhang Y, et al. ComBat-seq: batch effect adjustment for RNA-seq count data. *NAR Genom Bioinform*. 2020;2(3):lqaa078.

17. Subramanian A, et al. Gene set enrichment analysis: A knowledge-based approach for interpreting genome-wide expression profiles. *Proceedings of the National Academy of Sciences*. 2005;102(43):15545.

18. Hanzelmann S, et al. GSVA: gene set variation analysis for microarray and RNA-seq data. *BMC Bioinformatics*. 2013;14:7.

19. Salmon M, et al. KRAS4A induces metastatic lung adenocarcinomas in vivo in the absence of the KRAS4B isoform. *Proc Natl Acad Sci U S A*. 2021;118(30):e2023112118.

SUPPLEMENTAL FIGURES

Supplemental Figure 1

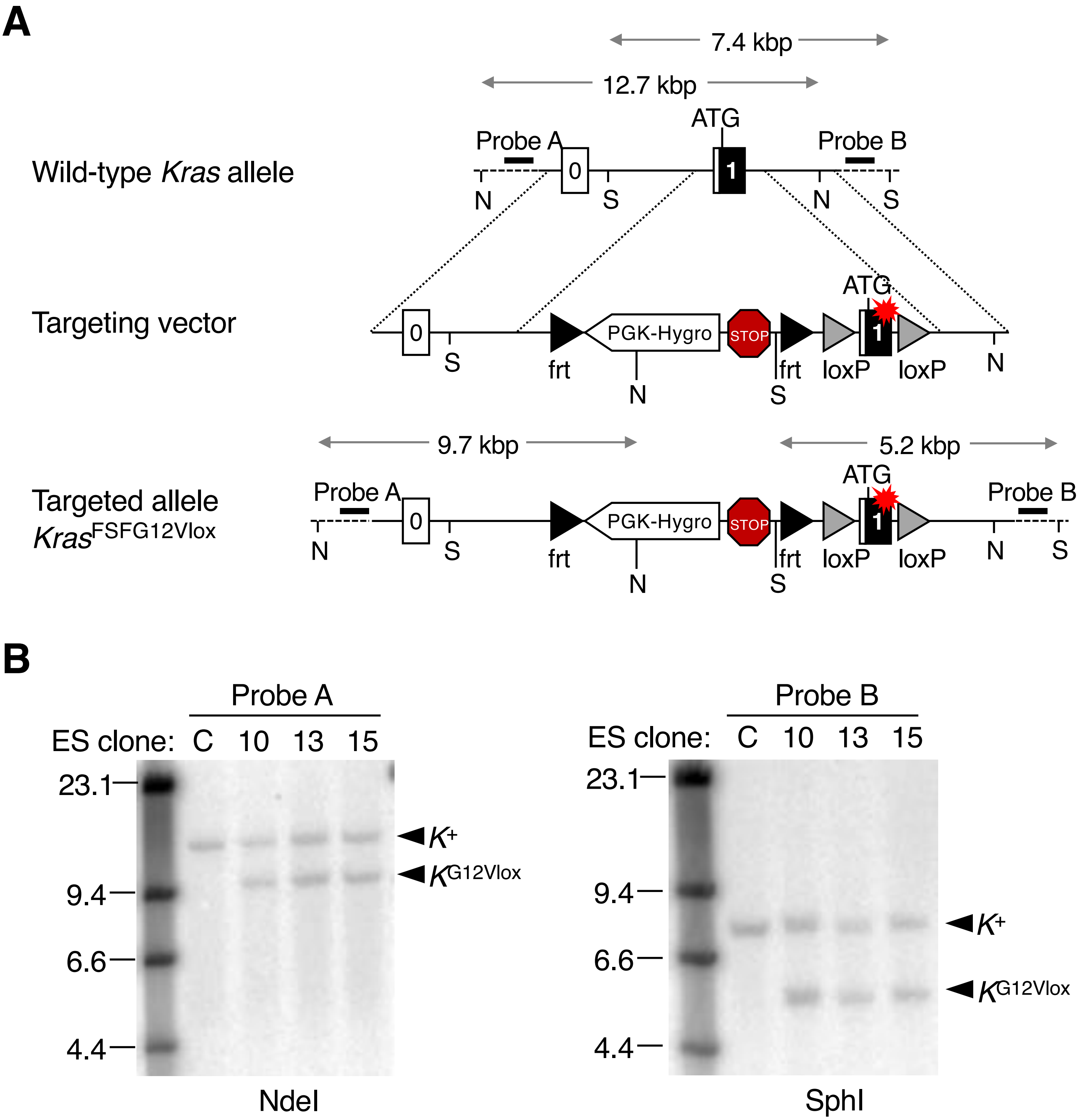

SUPPLEMENTAL FIGURE 1. Generation of a conditional (floxed) *Kras*$^{\mathbf{FSFG12Vlox}}$ oncogenic allele. (A) Gene targeting strategy: *Kras* exons 0 and 1 are indicated by open (non-coding sequences) and solid (coding sequences) boxes. The PGK-hygromycin resistance cassette (open arrow), transcriptional termination sequences (STOP, red octagonal box), *frt* sequences (solid triangles) and *loxP* sequences (grey triangles) are represented. The G12V mutation is displayed as a red star over exon 1. The ATG initiator codon is also shown. The position of probes A and B used for Southern blot analysis and restriction enzyme cleavage sites (N, NdeI; S, SphI) are also indicated. The diagnostic NdeI and SphI DNA fragments for the wild-type (12.7 and 7.4 kbp, respectively) and targeted (9.7 and 5.2 kbp, respectively) alleles are represented by arrows. **(B)** Southern blot analysis of DNA isolated from recombinant ES cell clones carrying wild-type (K^{+}) and targeted ($K^{G12Vlox}$) alleles. Migration of the diagnostic NdeI DNA fragments identified with Probe A (left) or Probe B (right) for the wild-type (K^{+}) and the targeted ($K^{G12Vlox}$) alleles are indicated by arrowheads. Fragments of DNA from bacteriophage λ digested with HindIII used for size determination are also shown.

Supplemental Figure 2

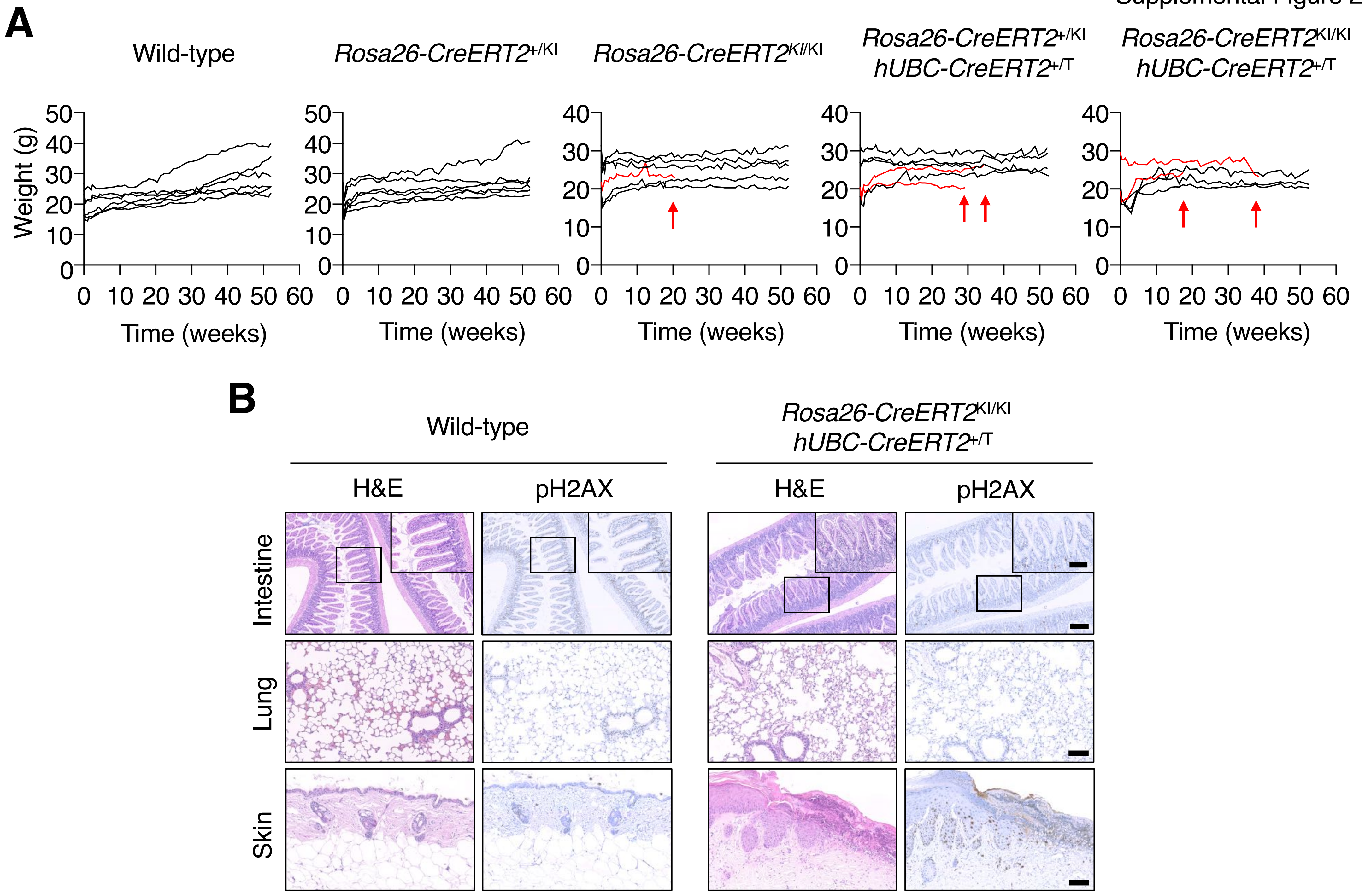

SUPPLEMENTAL FIGURE 2. CreERT2-associated toxicity in mice. (A) Body weight of mice harboring *Rosa26-CreERT2*^{KI} and *hUBC-CreERT2*^{T} alleles expressing the inducible CreERT2 recombinases systemically and exposed to TMX for one year. Genotypes of each group are depicted above each graph. Prematurely sacrificed mice are indicated by red lines. Red arrows indicate the time of death. Males and females are included in the experiment. **(B)** Representative images of H&E and phospho-H2AX (pH2AX) staining in paraffin-embedded sections of different tissues from control mice (*Rosa26-CreERT2*$^{+/+}$ and *hUBC-CreERT2*$^{+/+}$) and mice carrying three CreERT2 alleles (*Rosa26-CreERT2*$^{KI/KI}$ and *hUBC-CreERT2*$^{+/T}$) after one year of TMX exposure. Scale bar of intestine, low magnification 200 μm, and high magnification 100 μm; scale bar for the rest of tissues, 100 μm.

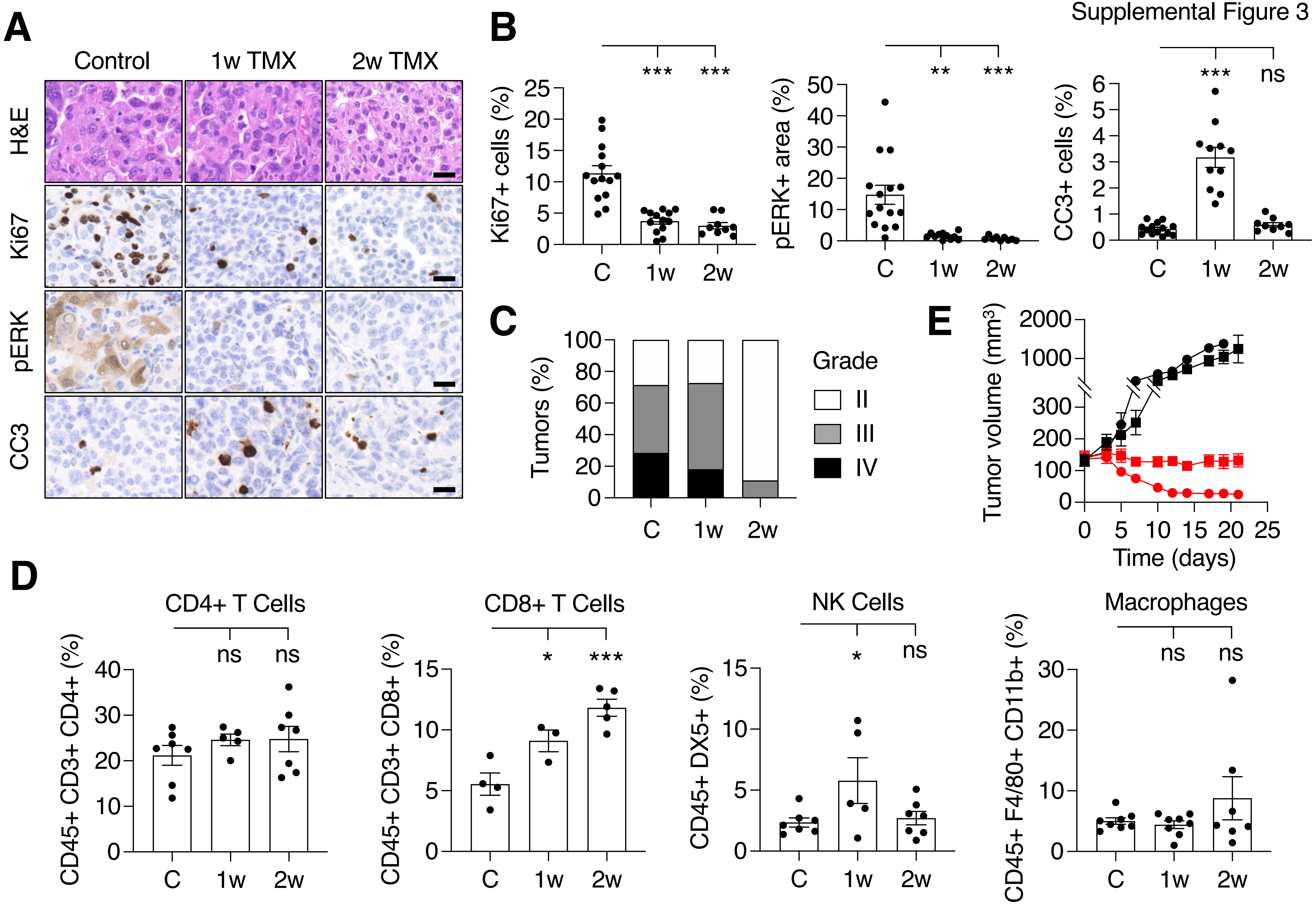
A
Control
1w TMX
2w TMX
H&E
Ki67
pERK
CC3
B
Ki67+ cells (%)
***
***
C
1w
2w
pERK+ area (%)
**
***
CC3+ cells (%)
***
ns
C
Tumors (%)
Grade
II
III
IV
E
Tumor volume (mm3)
Time (days)
D
CD4+ T Cells
CD45+ CD3+ CD4+ (%)
ns
ns
CD8+ T Cells
CD45+ CD3+ CD8+ (%)
*
***
NK Cells
CD45+ DX5+ (%)
*
ns
Macrophages
CD45+ F4/80+ CD11b+ (%)
ns
ns

SUPPLEMENTAL FIGURE 3. Tumor regression induced by *Kras*^{G12Vlox} ablation in K^{G12Vlox}PC2 mice. (A) Representative images of H&E, Ki67, pERK and cleaved caspase 3 (CC3) staining in paraffin-embedded sections of tumors from K^{G12Vlox}PC2 mice either untreated (Control) or exposed to a TMX diet for one (1w TMX) or two (2w TMX) weeks. Scale bar, 20 µm. **(B)** Quantification of the data shown in (A) as percentage of Ki67+, CC3+ cells and pERK+ area present in sections of tumors from K^{G12Vlox}PC2 mice either untreated (C) or treated with TMX for one (1w) or two (2w) weeks. Error bars indicate mean±SEM. *P* values were calculated using One-way ANOVA. **P < 0.01, ***P < 0.001, and ns, not significant. **(C)** Percentage of tumors of different histological grades (II to IV) present in lung sections of K^{G12Vlox}PC2 mice either untreated (C) or exposed to TMX diet for one (1w) or two (2w) weeks. **(D)** Flow cytometry analysis of infiltrating CD4+ T cells, CD8+ T cells, NK cells and macrophages in tumors from K^{G12Vlox}PC2 mice either untreated (C) or exposed to a TMX diet for one (1w) or two (2w) weeks, shown as percentage of the CD45+ gate. Error bars indicate mean±SEM. *P* values were calculated using One-way ANOVA. *P < 0.05, ***P < 0.001, ns, not significant. **(E)** Subcutaneous growth of two independent cell lines obtained from tumors grown in K^{G12Vlox}PC2 mice (circles and squares) in athymic NU-Foxn1^{nu} mice. Once the cell lines formed visible tumors, mice were either left untreated (black symbols) or exposed to TMX diet for the indicated time to excise the conditional *Kras*^{G12Vlox} alleles (red symbols) (n=6). Error bars indicate mean±SEM.

Supplemental Figure 4

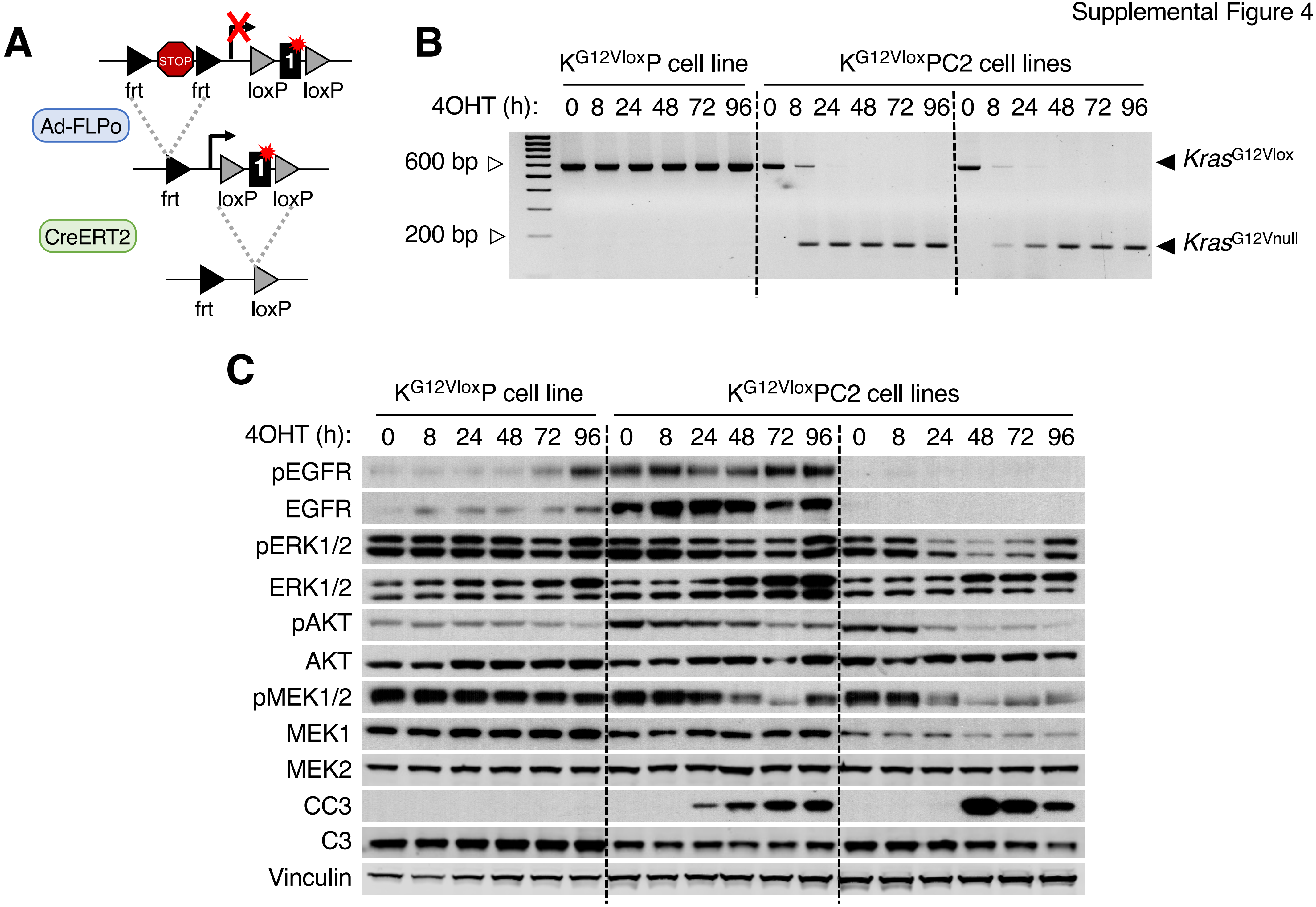

SUPPLEMENTAL FIGURE 4. Protein expression changes upon *Kras*^{G12Vlox} ablation in tumor cell cultures. (A) Schematic representation of the dual recombinase system used for the inducible expression of the *Kras*^{G12Vlox} allele and subsequent cleavage of its first exon to generate a *Kras*^{G12Vnull} allele. Top: Diagram of the *Kras*^{FSFG12Vlox} allele. Exon 1 (solid box) carrying the G12V mutation (red star), *frt* sites (black triangles), *loxP* sites (grey triangles) and transcriptional stop cassette (STOP) are represented in the scheme. Middle: Inducible expression of *Kras*^{G12Vlox} by Ad-FLPo-mediated recombination of the STOP cassette flanked by *frt* sequences. Bottom: *Kras*^{G12Vlox} ablation by TMX-inducible CreERT2 recombinases encoded by the *Rosa26-CreERT2*^{KI} allele and the *hUBC-CreERT2* transgene. **(B)** PCR analysis to identify the presence of the *Kras*^{G12Vlox} and *Kras*^{G12Vnull} alleles using genomic DNA from tumor cell lines derived from K^{G12Vlox}P and K^{G12Vlox}PC2 mice maintained in 4-hydroxytamoxifen (4-OHT)-containing medium for the indicated time. **(C)** Western blot analysis of phospho-EGFR, EGFR, phospho-ERK1/2, ERK1/2, phospho-AKT, AKT, phospho-MEK1/2, MEK1, MEK2, cleaved caspase 3 (CC3) and caspase 3 (C3) expression in lysates from K^{G12Vlox}P and K^{G12Vlox}PC2 tumor cell lines maintained in 4-OHT-containing medium for the indicated time. Vinculin served as loading control.

Supplemental Figure 5

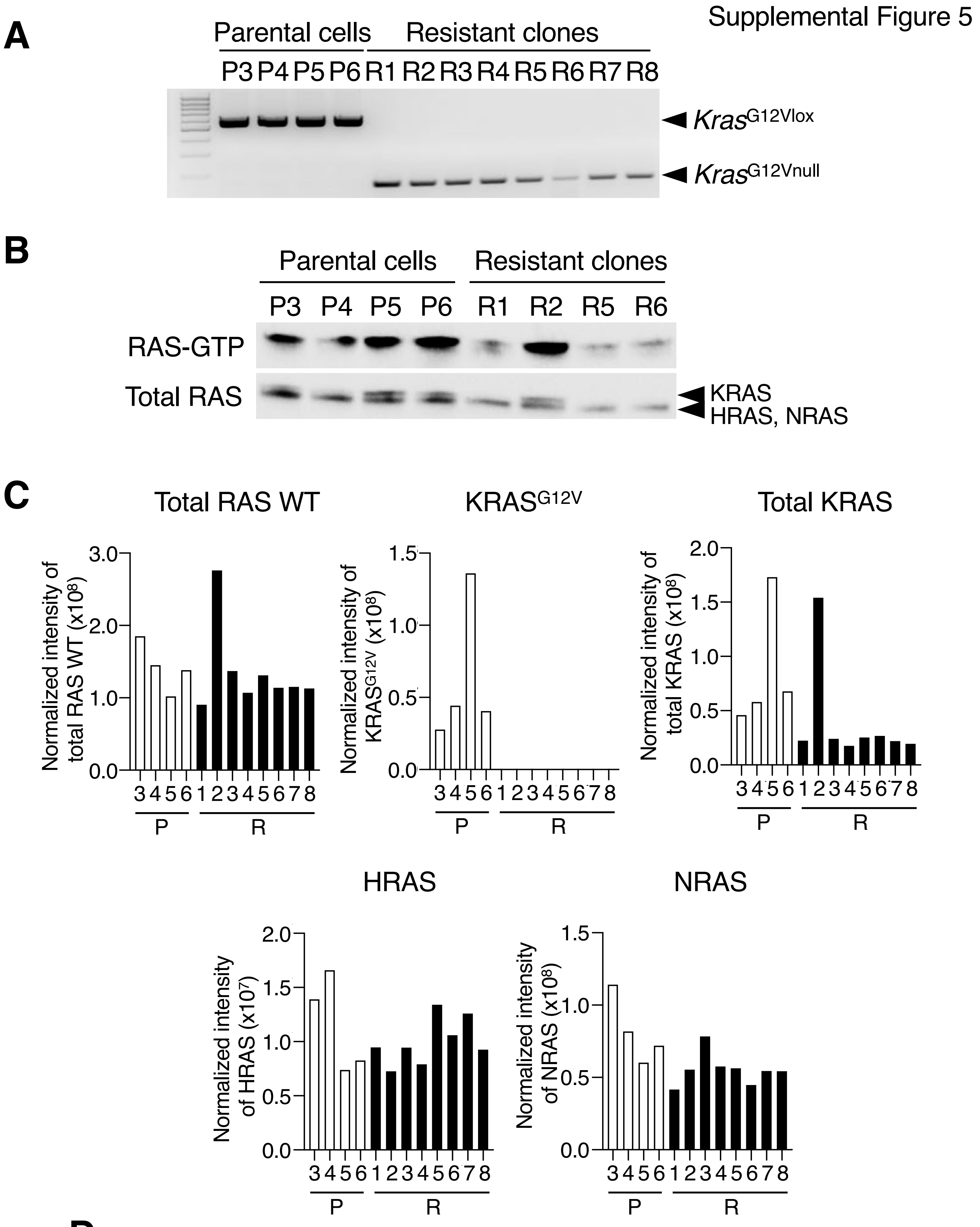

D

```
HRAS        MTEYKLVVVGAGGVGKSALTIQLIQNHFVDEYDPTIEDSYRKQVVIDGETCLLDILDTAG   60
NRAS        MTEYKLVVVGAGGVGKSALTIQLIQNHFVDEYDPTIEDSYRKQVVIDGETCLLDILDTAG   60
KRAS        MTEYKLVVVGAGGVGKSALTIQLIQNHFVDEYDPTIEDSYRKQVVIDGETCLLDILDTAG   60
KRASG12V    MTEYKLVVVGAVGVGKSALTIQLIQNHFVDEYDPTIEDSYRKQVVIDGETCLLDILDTAG   60
            *********** ************************************************

HRAS        QEEYSAMRDQYMRTGEGFLCVFAINNTKSFEDIHQYREQIKRVKDSDDVPMVLVGNKCDL   120
NRAS        QEEYSAMRDQYMRTGEGFLCVFAINNSKSFADINLYREQIKRVKDSDDVPMVLVGNKCDL   120
KRAS        QEEYSAMRDQYMRTGEGFLCVFAINNTKSFEDIHHYREQIKRVKDSEDVPMVLVGNKCDL   120
KRASG12V    QEEYSAMRDQYMRTGEGFLCVFAINNTKSFEDIHHYREQIKRVKDSEDVPMVLVGNKCDL   120
            **************************:*** **: ***********:*************
```

SUPPLEMENTAL FIGURE 5. Generation of tumor cell clones resistant to *Kras*$^{\mathrm{G12Vlox}}$ ablation. **(A)** PCR analysis of uncleaved *Kras*$^{\mathrm{G12Vlox}}$ and cleaved *Kras*$^{\mathrm{G12Vnull}}$ alleles using genomic DNA from parental cell lines (*Kras*$^{+/\mathrm{G12Vlox}}$;*Trp53*$^{-/-}$) and *Kras* resistant clones (*Kras*$^{+/-}$;*Trp53*$^{-/-}$). **(B)** Western blot analysis of activated RAS proteins (RAS-GTP) and total RAS proteins in whole cell lysates of parental cells and resistant clones using pan RAS antibodies. Migration of KRAS, HRAS and NRAS is indicated by arrows. **(C)** Relative quantification of the expression levels of total RAS, KRAS$^{\mathrm{G12V}}$, total KRAS, HRAS and NRAS proteins by label-free quantification in parental cell lines (P, open bars) and resistant clones (R, solid bars). **(D)** Protein sequence (amino acids 1-120) of HRAS, NRAS, KRAS and KRAS$^{\mathrm{G12V}}$, and the peptide sequences used for mass spectrometric analysis. Common HRAS, NRAS and KRAS peptides are marked in green, the KRAS$^{\mathrm{G12V}}$ specific peptide is colored in red, the unique peptides for HRAS are represented in orange, for NRAS in yellow and for KRAS in blue.

Supplemental Figure 6

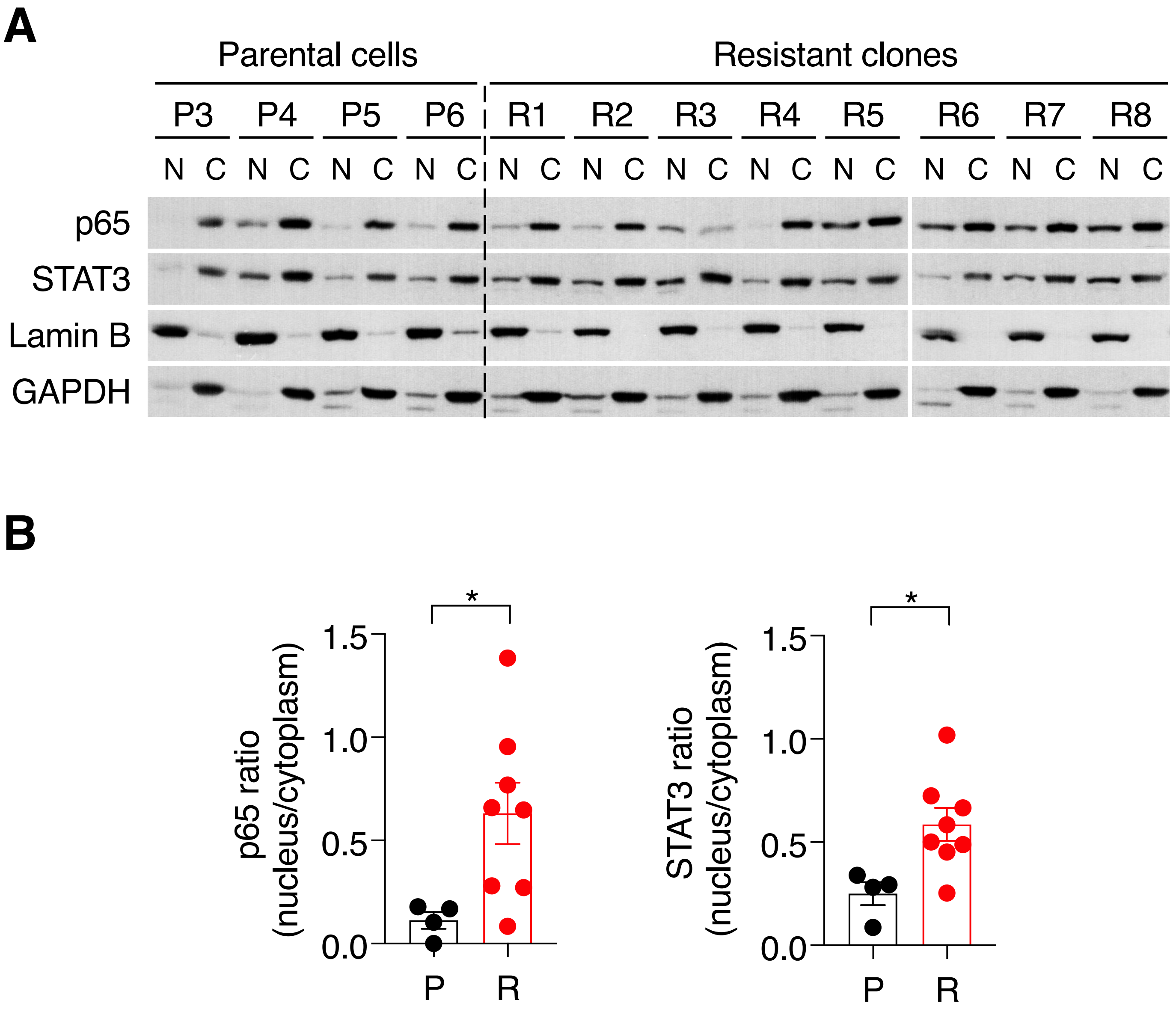

SUPPLEMENTAL FIGURE 6. Analysis of p65 and STAT3 expression in nuclear and cytoplasmic fractions of parental *Kras*$^{+/G12Vlox}$;*Trp53*$^{-/-}$ cell lines and resistant *Kras*$^{+/-}$;*Trp53*$^{-/-}$ clones. (A) Western blot analysis of p65 and STAT3 expression in nuclear and cytoplasmic fractions from parental *Kras*$^{+/G12Vlox}$;*Trp53*$^{-/-}$ cell lines (P3 to P6) and resistant *Kras*$^{+/-}$;*Trp53*$^{-/-}$ clones (R1 to R8). GAPDH was used as a cytoplasmic (C) marker protein and Lamin B as a marker of the nuclear fraction (N). **(B)** Quantification of the nuclear/cytoplasmic ratio of p65 and STAT3 from the western blot images described in (A) in parental *Kras*$^{+/G12Vlox}$;*Trp53*$^{-/-}$ cells (P, black) and resistant *Kras*$^{+/-}$;*Trp53*$^{-/-}$ clones (R, red). Error bars indicate mean±SEM. *P* values were calculated using the unpaired Student's t test. *P < 0.05.

A

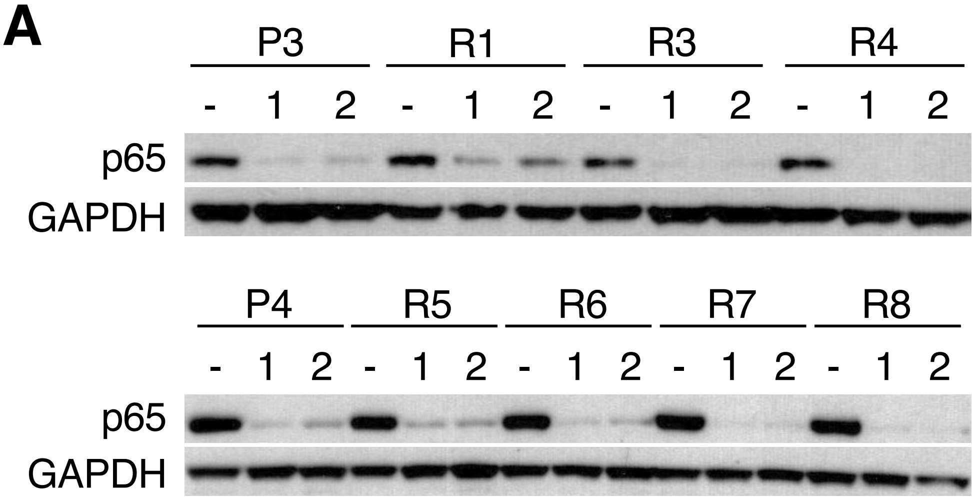
P3
R1
R3
R4
- 1 2 - 1 2 - 1 2 - 1 2
p65
GAPDH
P4
R5
R6
R7
R8
- 1 2 - 1 2 - 1 2 - 1 2 - 1 2
p65
GAPDH

B

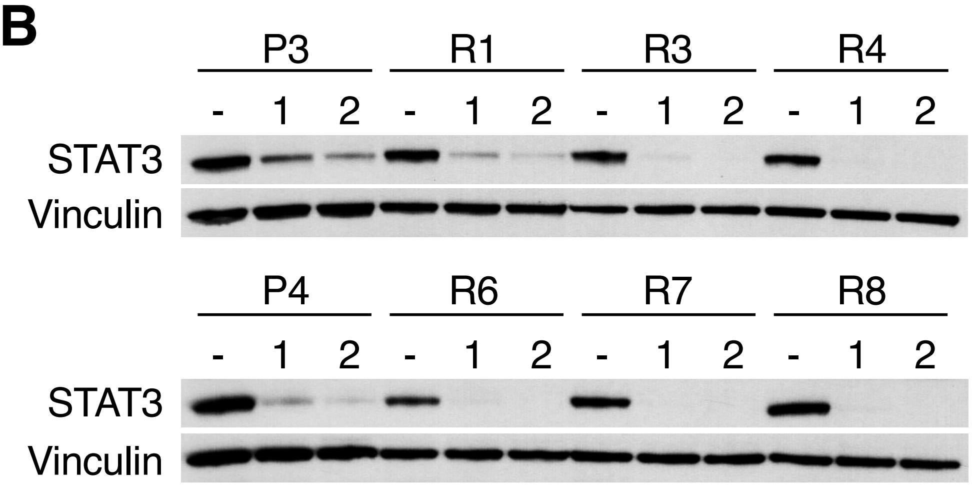
P3
R1
R3
R4
- 1 2 - 1 2 - 1 2 - 1 2
STAT3
Vinculin
P4
R6
R7
R8
- 1 2 - 1 2 - 1 2 - 1 2
STAT3
Vinculin

SUPPLEMENTAL FIGURE 7. Validation of independent shRNAs against p65 and STAT3. **(A)** Western blot analysis of p65 expression in lysates from parental cell lines (P3, P4) and clones resistant to *Kras*^{G12Vlox} ablation (R1, R3, R4, R6, R7 and R8) upon incubation with two independent shRNAs (#1, TRCN0000055346; and #2, TRCN0000235832). GAPDH served as loading control **(B)** Western blot analysis of STAT3 expression in the lysates described above upon incubation with two independent shRNAs (#1, TRCN0000071453; and #2, TRCN0000071454). Vinculin served as loading control.

Supplemental Figure 8

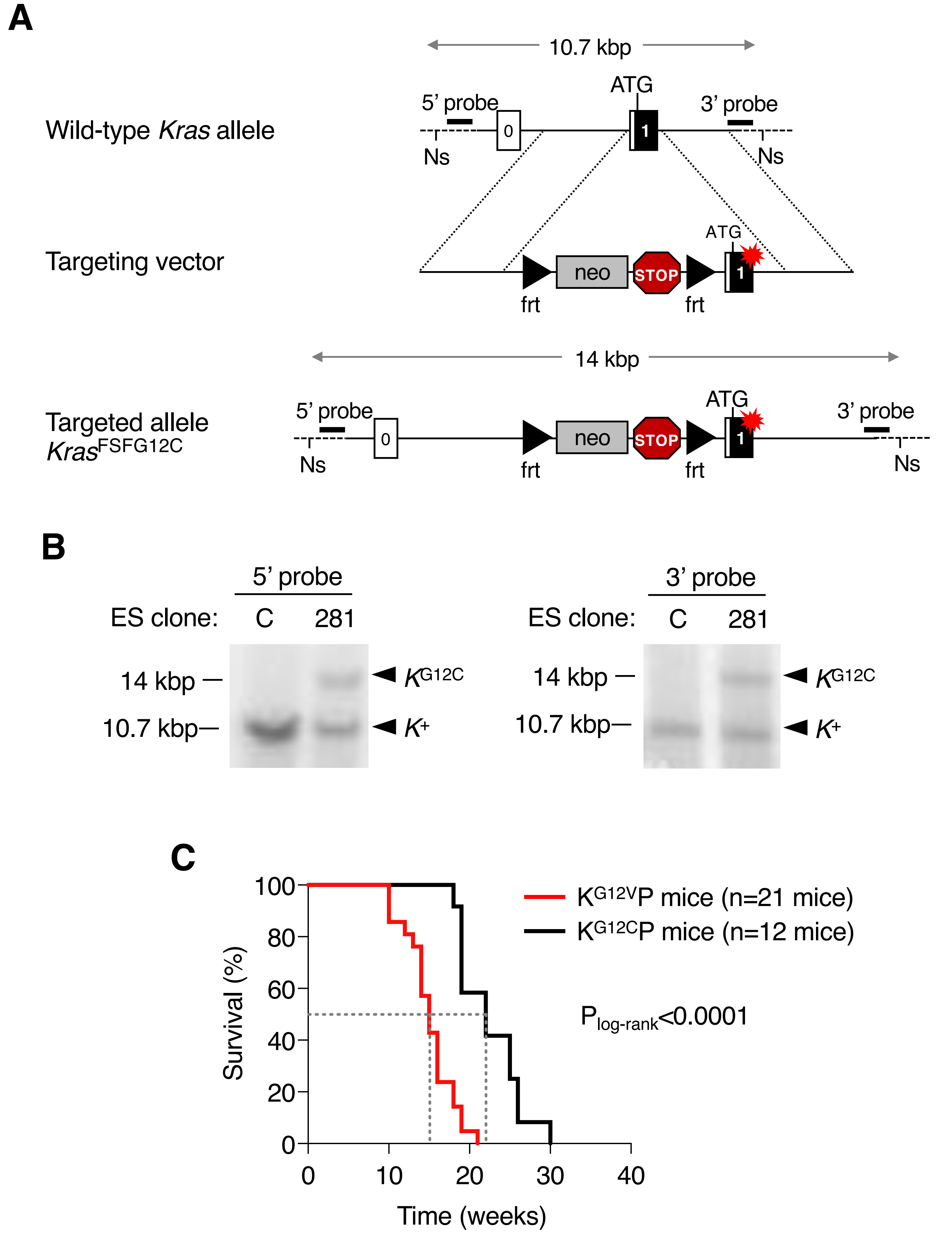

SUPPLEMENTAL FIGURE 8. Generation of a conditional knock-in mouse model expressing the KRAS^{G12C} oncoprotein. **(A)** Gene targeting strategy: *Kras* exons 0 and 1 are indicated by open (non-protein coding sequences) and solid boxes (protein-coding sequences). The neomycin resistance cassette (grey box), transcriptional termination sequences (STOP, red octagonal box) and *frt* sequences (black triangles) are represented. The G12C mutation is indicated as a red star over exon 1. The ATG initiator codon is also shown. The position of the 5' and 3' probes used for Southern blot analysis and the NsiI restriction enzyme cleavage sites (Ns) are also indicated. The diagnostic NsiI DNA fragments for the wild-type (10.7 kbp) and targeted alleles (14 kbp) are represented by arrows. **(B)** Southern blot analysis of DNA isolated from recombinant ES cell clones carrying wild-type (K^{+}) and recombinant alleles (K^{G12C}). The sizes of the diagnostic NsiI DNA fragments are indicated by arrowheads. **(C)** Survival of *Kras*$^{+/FSFG12V}$;*Trp53*$^{F/F}$ (red, n=21 mice) and *Kras*$^{+/FSFG12C}$;*Trp53*$^{F/F}$ (black, n=12 mice) mice infected with Adeno-FLPo. $P_{log\text{-}rank} < 0.0001$.

Chromosome 6: Control tumors

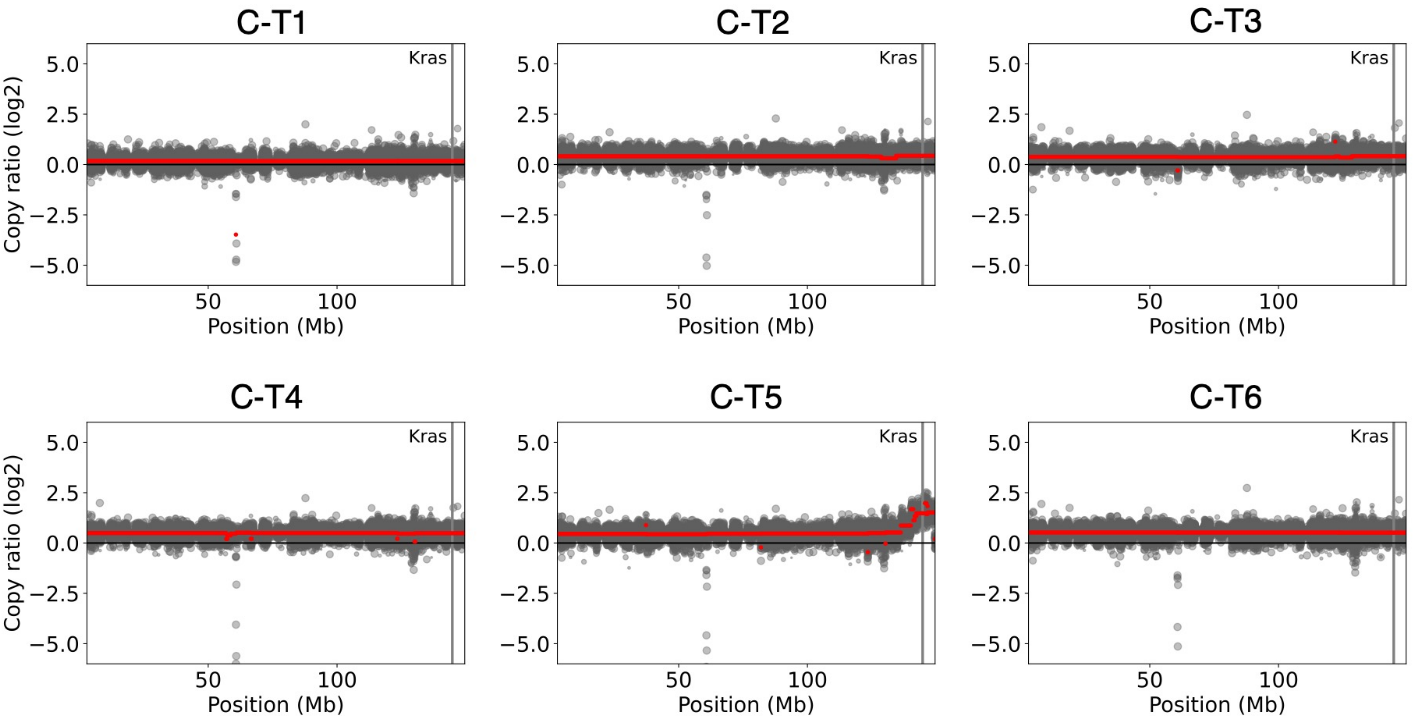

Chromosome 6: Sotorasib-resistant tumors

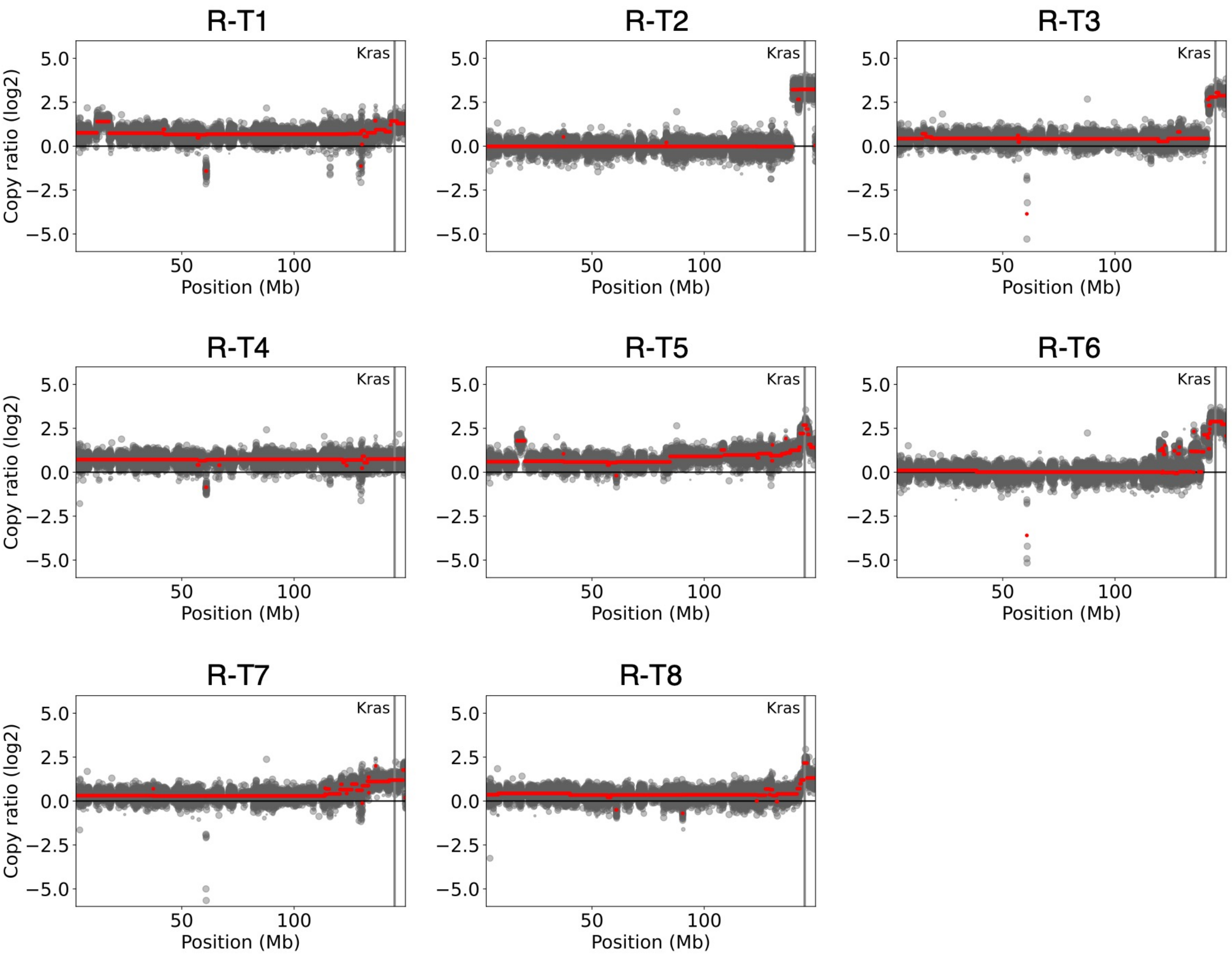

SUPPLEMENTAL FIGURE 9. DNA copy number variations (CNVs) in chromosome 6 of control and sotorasib-resistant lung tumors. CNVs are displayed as scatter plots of bin-level log2 coverages with overlaid segments of the selected chromosomal region. *Kras* is highlighted with a vertical line. The size of the plotted datapoints is proportional to the weight of each point used in segmentation.

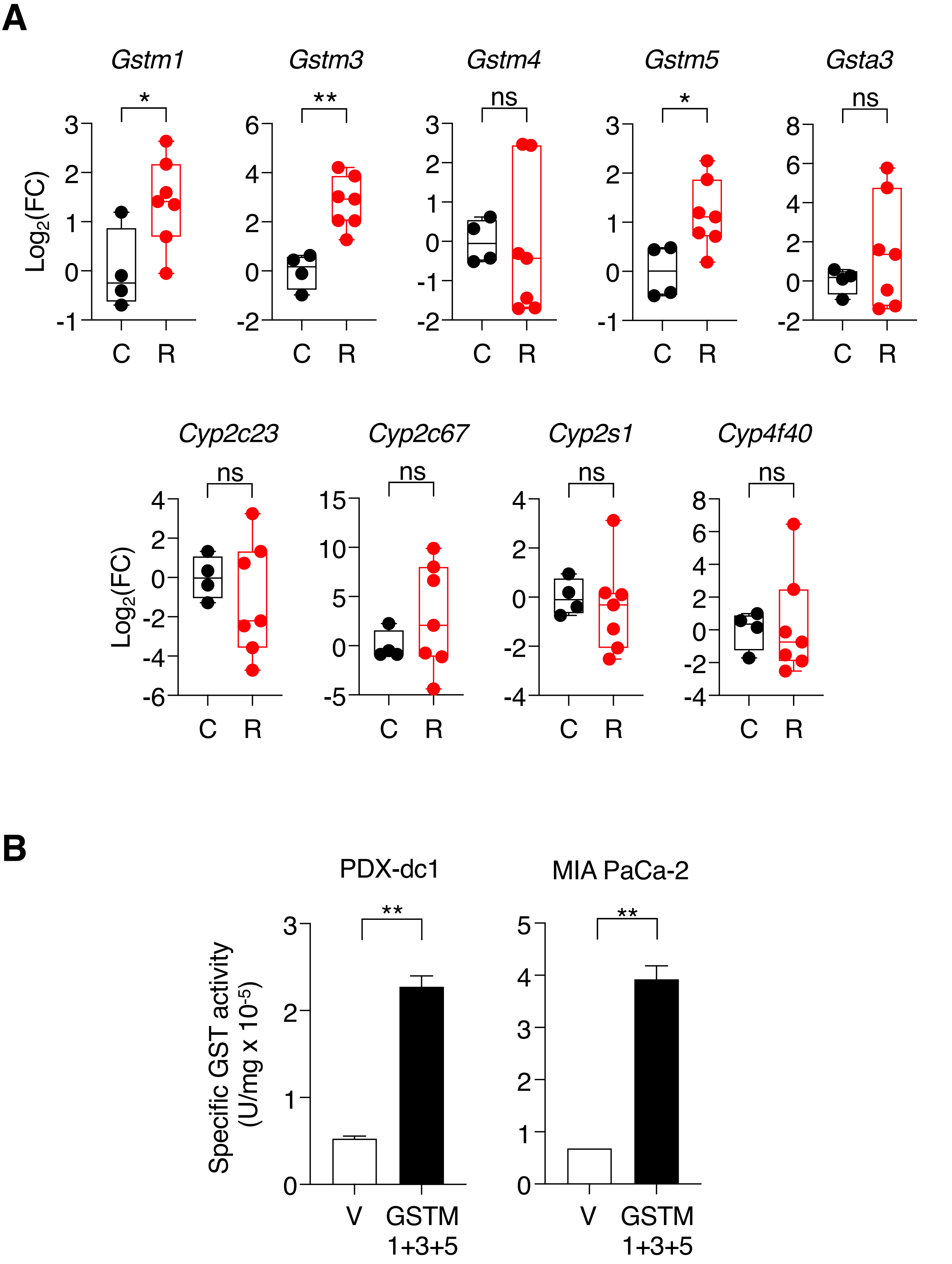
A
Gstm1
Gstm3
Gstm4
Gstm5
Gsta3
*
**
ns
*
ns
Log2(FC)
C R
Cyp2c23
Cyp2c67
Cyp2s1
Cyp4f40
ns
ns
ns
ns
B
PDX-dc1
MIA PaCa-2
**
**
Specific GST activity (U/mg x 10-5)
V
GSTM 1+3+5

SUPPLEMENTAL FIGURE 10. Validation of genes related to xenobiotic metabolism pathways in sotorasib-resistant tumor cells. (A) Log2 fold change (FC) values determined by qRT-PCR for xenobiotic metabolism-related genes in cell lines obtained from control tumors expressing *Kras*^{G12C} (C, black, n=4) and in cell lines obtained from sotorasib-resistant tumors expressing *Kras*^{G12C} cultured in the presence of 10 μM of sotorasib (R, red, n=7). β-actin was used for normalization. *P* values were calculated using the unpaired Student's t test. *P < 0.05 and **P < 0.01; ns, not significant. **(B)** Specific GST activity (U/mg) of PDX-dc1 and MIA PaCa-2 cells infected with empty lentiviral vectors (V) or lentiviral vectors expressing GSTM1, GSTM3 and GSTM5 (GSTM 1+3+5). *P* values were calculated using the unpaired Student's t test. **P < 0.01.

Supplemental Figure 11

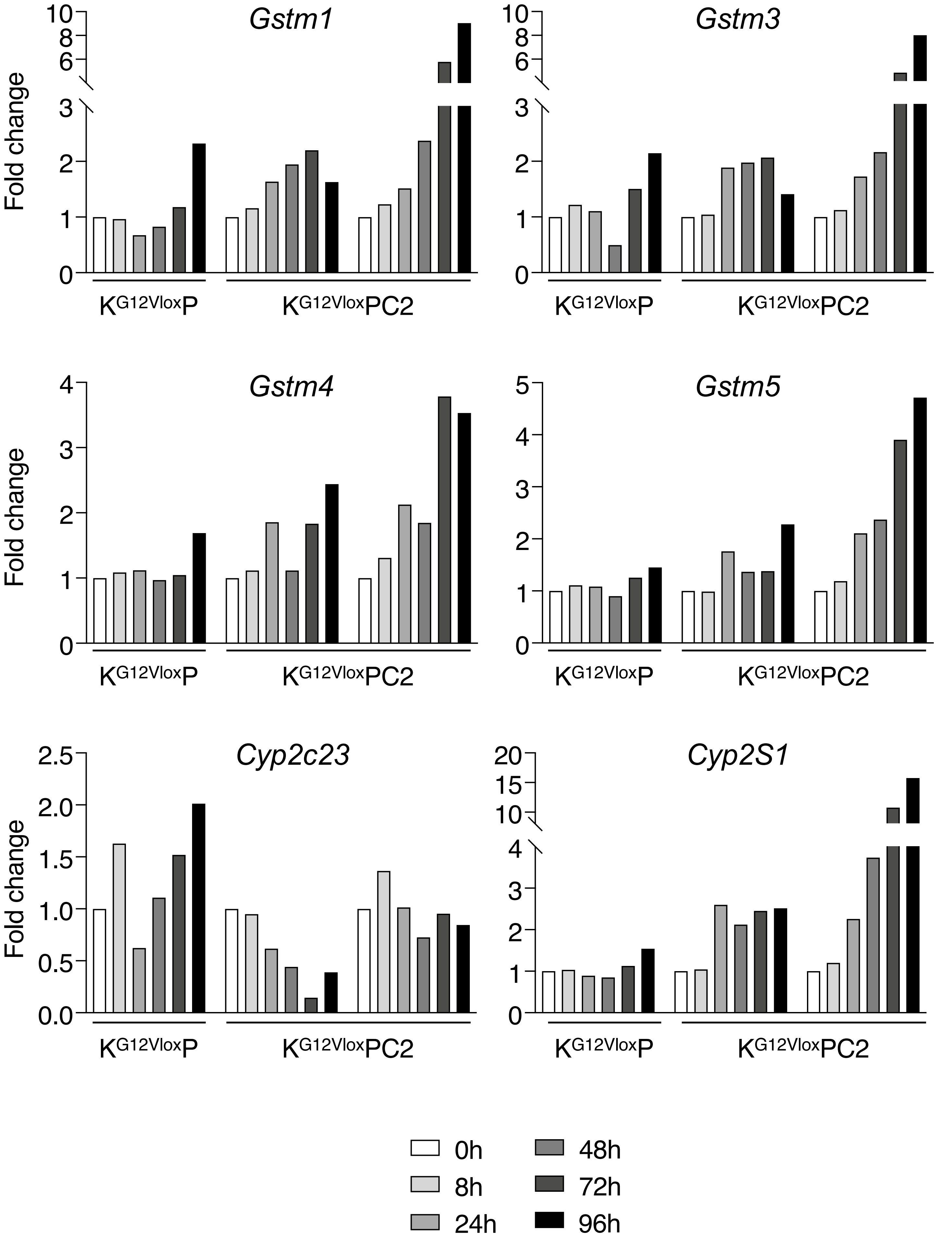

SUPPLEMENTAL FIGURE 11. Expression changes of xenobiotic metabolism-related genes upon *Kras*^{G12Vlox} ablation in tumor cell cultures. Fold change values determined by qRT-PCR for the indicated xenobiotic metabolism-related genes from a $K^{G12Vlox}P$ and two $K^{G12Vlox}PC2$ tumor cell lines maintained in 4-hydroxytamoxifen-containing medium for the indicated times. β-actin was used for normalization.

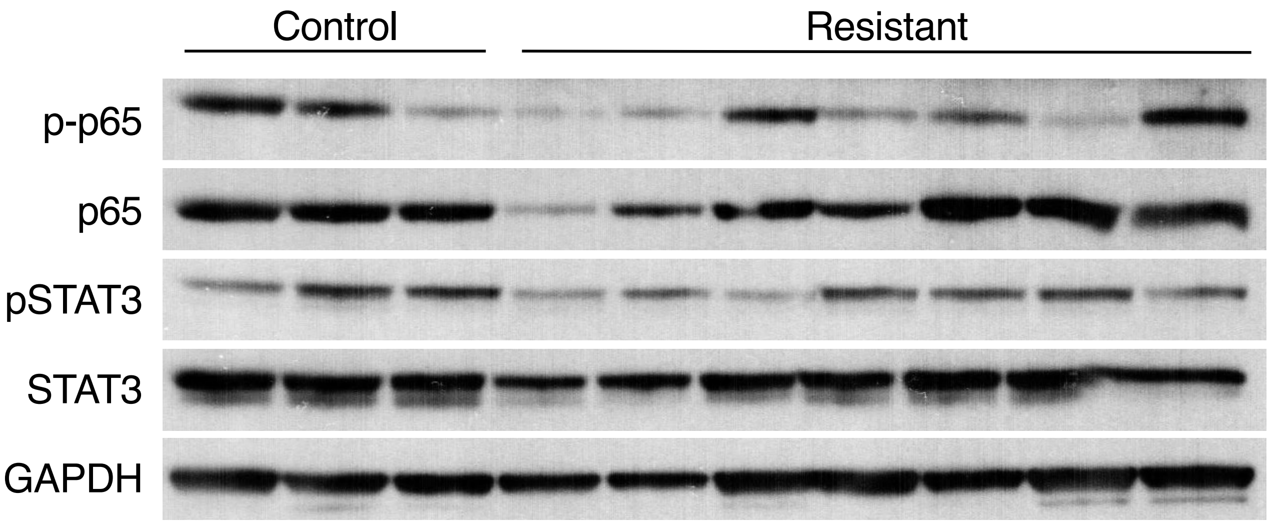
Control
Resistant
p-p65
p65
pSTAT3
STAT3
GAPDH

SUPPLEMENTAL FIGURE 12. Analysis of NF-κB and STAT3 signaling pathways in control and sotorasib-resistant lung tumors. Western blot analysis of phospho-p65 (p-p65), p65, phospho-STAT3 (pSTAT3) and STAT3 expression in lysates from control and sotorasib-resistant tumors. GAPDH served as loading control.

Supplemental Figure 13

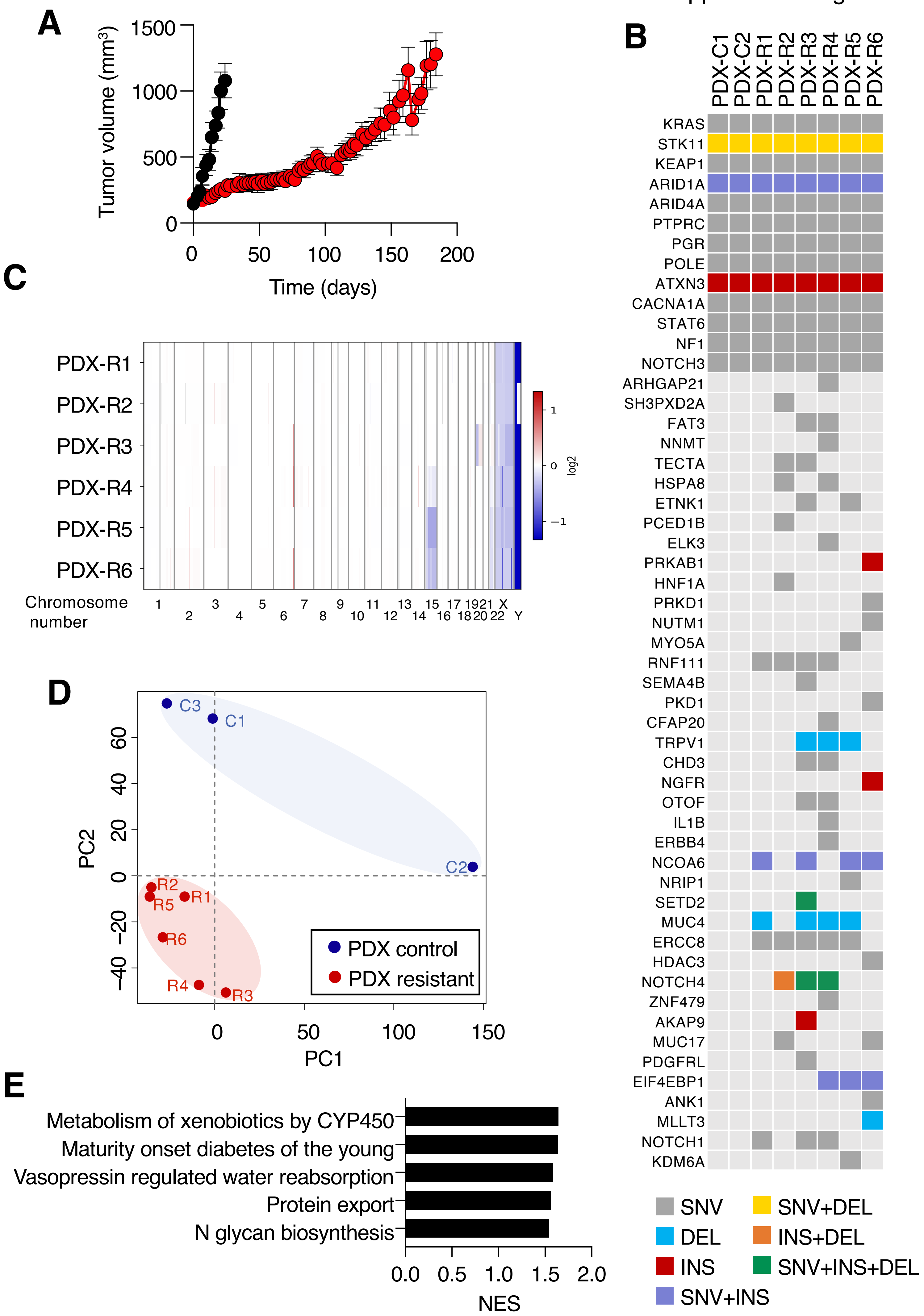

SUPPLEMENTAL FIGURE 13. Induction of sotorasib resistance in a Patient-derived xenograft (PDX). (A) Tumor growth of individual fragments of a PDX tumor model treated with vehicle (n=8, black) or with 100 mg/kg of sotorasib (n=6, red). Error bars indicate mean±SEM. **(B)** Representative mutations in genes described to be altered in LUAD identified by whole exome sequencing of two PDX replicates treated with vehicle (PDX-C1 and PDX-C2) and the six sotorasib-resistant PDX replicates (PDX-R1 to PDX-R6). Single nucleotide variants (SNV, dark grey), deletion (DEL, blue), insertion (INS, red), SNV and insertion (SNV+INS, purple), SNV and deletion (SNV+DEL, yellow), deletion and insertion (INS+DEL, orange), and SNV, deletion and insertion (SNV+DEL+INS, green) are also indicated. **(C)** Heatmap representing log2-ratio CNVs from whole exome sequencing data of sotorasib resistant PDX tumors in all chromosomes. Each row represents an individual sample. **(D)** PCA displaying the distribution of control (blue) and sotorasib-resistant (red) PDX tumors. **(E)** Normalized enrichment scores (NES) of biological pathways significantly enriched in sotorasib-resistant PDX tumors obtained from GSEA of KEGG gene sets. Only gene sets significantly enriched at False Discovery Rate (FDR) q-values < 0.25 where considered.
